# Transcriptome analysis highlights key differentially expressed genes involved in cellulose and lignin biosynthesis of sugarcane genotypes varying in fiber content

**DOI:** 10.1038/s41598-018-30033-4

**Published:** 2018-08-02

**Authors:** Lakshmi Kasirajan, Nam V. Hoang, Agnelo Furtado, Frederik C. Botha, Robert J. Henry

**Affiliations:** 1 Queensland Alliance for Agriculture and Food Innovation, The University of Queensland, St. Lucia, Queensland, 4072 Australia; 2 ICAR-Sugarcane Breeding Institute, Coimbatore, 641007 Tamil Nadu India; 3 College of Agriculture and Forestry, Hue University, Hue, Vietnam; 4 Sugar Research Australia, Indooroopilly, Queensland, 4068 Australia

## Abstract

Sugarcane (*Saccharum* spp. hybrids) is a potential lignocellulosic feedstock for biofuel production due to its exceptional biomass accumulation ability, high convertible carbohydrate content and a favorable energy input/output ratio. Genetic modification of biofuel traits to improve biomass conversion requires an understanding of the regulation of carbohydrate and lignin biosynthesis. RNA-Seq was used to investigate the transcripts differentially expressed between the immature and mature tissues of the sugarcane genotypes varying in fiber content. Most of the differentially expressed transcripts were found to be down-regulated during stem maturation, highlighting their roles in active secondary cell-wall development in the younger tissues of both high and low fiber genotypes. Several cellulose synthase genes (including CesA2, CesA4, CesA7 and COBRA-like protein), lignin biosynthesis-related genes (ρ-coumarate 3-hydroxylase, ferulate 5-hydroxylase, cinnamyl alcohol dehydrogenase and gentiobiase) and transcription regulators for the secondary cell-wall synthesis (including LIM, MYB, PLATZ, IAA24, C2H2 and C2C2 DOF zinc finger gene families) were exclusively differentially expressed between immature and mature tissues of high fiber genotypes. These findings reveal target genes for subsequent research on the regulation of cellulose and lignin metabolism.

## Introduction

The use of biofuels to satisfy energy demand relies upon the development and efficient supply of suitable biomass as feedstock^1–3^. C_4_ plants like *Miscanthus*, maize, sorghum, and sugarcane are naturally more photosynthetically efficient than C_3_ plants in biomass production in the tropical and temperate regions, thanks to their high water use efficiency and CO_2_-concentrating mechanism^4,5^. Among the high biomass producing crops, the panicoid grasses which are exclusively C_4_ are potential candidate feedstock for the production of lignocellulosic biofuel. Sugarcane (*Saccharum* spp. hybrids) has been identified as one of major lignocellulosic feedstock sources for biofuel production due to its exceptional biomass accumulation ability, high convertible carbohydrate content and a favorable energy input/output ratio^6–8^. Sugarcane bagasse is composed of 35–40% cellulose 25–30% hemicellulose, 20–25% lignin, 1–3% ash and 2–3% other components^9,10^, which are constituents of the secondary cell-wall of all vascular plants. Cellulose microfibrils are embedded in a matrix of hemicellulose molecules cross-linked with lignin polymers forming the skeleton of plants cells^11^. Not only glucose from cellulose, but also hemicellulose is a rich source of sugars for fermentation, but the complex interlinkage with lignin monomers makes the biomass recalcitrant and limits the release of fermentable sugars. Recalcitrance of the biomass is not only caused by the lignin content but also by its composition, namely, syringyl (lignin S), guaiacyl (lignin G) and p-hydroxyphenyl (lignin H)^12,13^.

Recently, genetic engineering of bioenergy crops has been used to produce plants with reduced lignin content or modified composition for easy hydrolysis and saccharification^14,15^. Knowledge of genes involved in the carbohydrate metabolism and phenylpropanoid pathways, which provides the precursors for cellulose and lignin biosynthesis, respectively, is necessary to explore the effects of biomass recalcitrance and improve the conversion efficiency of lignocellulosic feed stocks. Many research groups have identified and characterised the genes and transcription factors involved in biomass production, especially in the carbohydrate and phenylpropanoid pathways, in a number of plant species and tissues providing specific targets for the biomass modification^16–26^. However, knowledge of gene expression (hereafter used to mean expression of a transcript of the gene), regulation of polysaccharide and lignin metabolism in the developing sugarcane stems (especially of those contrasting genotypes for biomass traits) is limited with only a few genes identified through transcriptome profiling. In this present study, we used RNA-Seq analysis to compare the transcript profiles of the immature (forth internode from top) and mature (third internode from bottom) tissues of twenty sugarcane genotypes of high and low fiber, to highlight the gene expression differences between the tissue types from the two respective genotype groups. The current study focuses specifically on differentially expressed (DE) genes/transcripts that are involved in cellulose and lignin biosynthesis, to identify potential genes/transcriptions factor for sugarcane biomass modification, thereby reducing the cost of pre-treatment for biofuel production.

## Results

### Sample selection and RNA-Seq summary

The genetic material chosen for the study included twenty sugarcane genotypes, categorized into two different groups of 10 high and 10 low fiber genotypes, which consisted of equal numbers of commercial genotypes and introgression lines derived from crosses between wild *Saccharum spontaneum* relatives and *Erianthus* species. The phenotypic data for this collection was derived from a previous study^27^. In brief, the fiber content in these genotypes ranged from 8.4 to 15% of total fresh mass (total water and solid content which include sugars, fiber and other components of the sugarcane culm biomass); while the lignin content of the genotypes in the high fiber group was 3.0 to 3.4%, and the low fiber group, 2.0 to 2.5% of total fresh biomass (Table 1).

**Table 1:** Near-Infrared Spectroscopy predicted biomass composition of high and low fiber sugarcane genotypes selected for the study.

CODE^*^	Genotype	Type	Predicted values based on NIR^**^			
			Fiber in 400 g (%)	Glucose (%)	Hemicellulose (%)	Total lignin (%)
LF18	KQB09-23137	Introgression	8.4	3.7	2.7	2.0
LF1	QC02-402	Commercial	8.5	4.0	2.5	2.0
LF19	KQB09-20620	Introgression	9.3	4.4	2.8	2.1
LF10	QN05-803	Commercial	9.6	4.6	2.7	2.2
LF6	QS99-2014	Commercial	9.9	4.7	2.9	2.3
LF8	Q241	Commercial	10.0	4.9	2.8	2.2
LF13	KQB07-23990	Introgression	10.2	4.9	3.0	2.4
LF12	KQB08-32953	Introgression	10.2	4.8	3.0	2.4
LF4	QN05-1743	Commercial	10.9	5.1	3.3	2.5
LF11	KQB07-23863	Introgression	11.4	5.7	3.4	2.3
HF5	QN05-1509	Commercial	13.6	6.6	3.9	3.1
HF9	Q200	Commercial	13.7	6.7	4.0	3.1
HF20	KQB09-20432	Introgression	14.2	7.0	4.0	3.2
HF17	QBYN04-26041	Introgression	14.3	7.2	4.1	3.0
HF3	QN05-1460	Commercial	14.4	6.8	4.3	3.3
HF15	KQB07-24619	Introgression	14.5	6.7	4.4	3.4
HF14	KQ08-2850	Introgression	14.6	7.1	4.4	3.1
HF16	KQB07-24739	Introgression	14.7	7.0	4.3	3.4
HF7	QA96-1749	Commercial	14.8	6.9	4.5	3.4
HF2	QA02-1009	Commercial	15.0	7.3	4.3	3.4

^*^LF denotes low Fiber, HF denotes High fiber. ^**^Data expressed as percentage of total fresh mass.

Transcriptome sequencing was carried out for top and bottom internodal samples of ten high and ten low fiber sugarcane genotypes at 12^th^ months of age, using the extracted RNA samples with a RIN number of 7.5 and above (see Methods, additional File 6), to study the differential expression pattern of cellulose and lignin genes. The average length of the RNA-Seq reads was 116 and 128 bp after pre-processing for top and bottom tissues, respectively. After stringent adapter trimming and quality filtering, the cleaned data with a Phred score of 20 and greater were aligned to the *Saccharum officinarum* gene indices (SoGI) v3.0^28^ using RNA-Seq analysis tool in CLC Genomics Workbench (CLC-GWB) with length fraction 0.9 and similarity fraction of 0.8. The total number of trimmed reads for all the samples from the low fiber group was 731 million reads, while that of the high fiber group was 778 million reads. This made the total read data used in this analysis, 1,509 million reads. The number of raw reads, trimmed reads and their mapping percentage against the SoGI reference database obtained for all of these 40 samples are shown in Table 2. The percentage of reads mapped to the transcriptome reference ranged from 44.3% to 56%.

**Table 2:** Summary of RNA-Seq mapping results of high and low fiber genotypes against the *Saccharum officinarum* gene index database.

Low fiber genotypes						
Sample	Bottom Internode (reads)			Top Internode (reads)		
	Raw data	Trimmed data	Total mapped with SoGI	Raw data	Trimmed data	Total mapped with SoGI
LF 1	41,184,328	41,184,328	20,697,189 (50.25%)	19,229,242	19,224,245	8,884,192 (48.21%)
LF4	22,875,506	22,869,941	10,133,465 (44.30%)	24,841,374	24,837,412	12,420,765 (50.00%)
LF6	24,972,168	24,967,893	11,458,609 (45.893%)	12,975,482	12,971,379	6,531,392 (50.35%)
LF8	53,221,280	53,217,475	27,956,352 (52.53%)	28,678,158	28,674,721	14,239,781 (49.65%)
LF10	80,816,900	80,811,427	43,123,776 (53.36%)	15,283,482	15,280,984	7,337,053 (48.01%)
LF11	50,927,916	50,924,128	26,693,455 (52.41%)	8,693,292	8,692,076	4,237,167 (48.74%)
LF12	63,806,938	63,803,940	34,091,070 (53.43%)	52,857,488	52,848,075	26,332,776 (49.86%)
LF13	24,201,428	24,196,385	13,435,469 (55.52%)	19,445,552	19,443,531	9,638,645 (49.57%)
LF18	66,637,338	66,628,598	34,753,721 (52.16%)	64,795,122	64,789,858	33,653,146 (51.94%)
LF19	30,957,686	30,954,783	16,694,345 (53.93%)	24,885,118	24,882,334	11,212,753 (45.06%)
TOTAL	459,601,488	459,558,898	239,037,451 (52.02%)	271,684,310	271,644,615	134,487,670 (49.51%)
**High fiber genotypes**						
	**Bottom Internode (reads)**			**Top Internode (reads)**		
HF 2	79,764,754	79,760,012	42,139,460 (52.83%)	42,730,848	42,714,286	18,661,601 (43.68%)
HF3	140,689,668	140,683,201	73,590,904 (52.30%)	22,661,194	22,656,506	10,316,204 (45.53%)
HF5	47,980,196	47,973,820	22,930,433 (47.79%)	8,580,356	8,578,357	4,023,865 (46.90%)
HF7	23,697,632	23,694,629	11,458,836 (48.36%)	25,210,536	25,204,740	12,510,770 (49.63%)
HF9	36,369,116	36,366,607	18,882,822 (51.92%)	36,823,962	36,810,635	18,112,124 (49.20%)
HF14	19,179,058	19,172,922	9,388,004 (48.96%)	37,855,198	37,845,062	18,700,773 (49.41%)
HF15	14,564,738	14,562,171	6,819,730 (46.83%)	49,037,414	49,028,202	25,086,777 (51.16%)
HF16	46,041,750	46,037,690	24,256,933 (52.69%)	28,878,114	28,875,842	14,202,010 (49.18%)
HF17	50,934,370	50,930,566	26,722,984 (52.47%)	15,537,556	15,535,545	7,970,199 (51.30%)
HF20	45,505,900	45,500,054	24,074,451 (52.91%)	6,531,654	6,529,582	3,096,386 (47.42%)
TOTAL	504,727,182	504,681,672	260,264,557 (51.57%)	273,846,832	273,778,757	132,680,709 (48.46%)

### Global expression analysis of sugarcane RNA-Seq data

**Figure 1:**
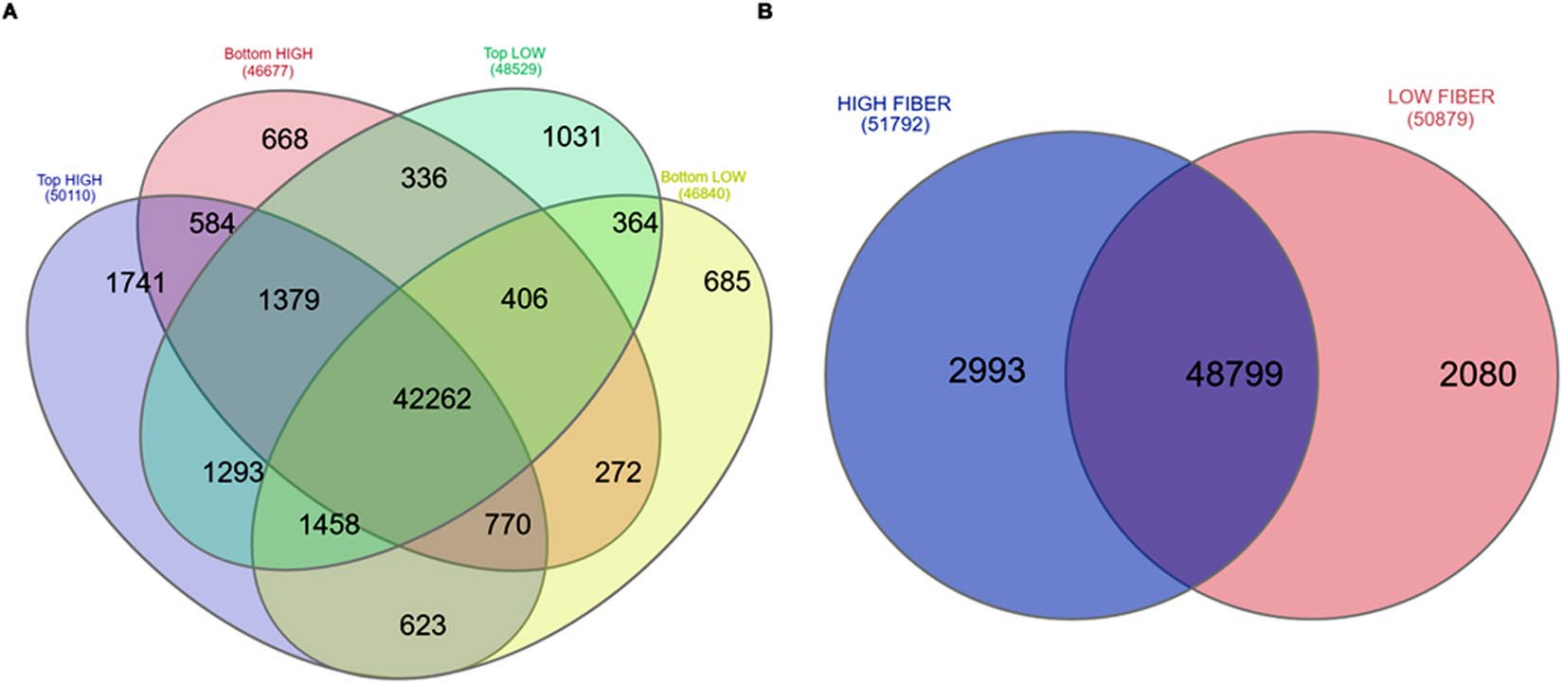
Venn diagram of global expression analysis of internodal samples used in this study. **(A)** Comparison of SoGI sequences expressed in top and bottom internodes of high and low fiber genotypes with SoGI as reference database. (**B**) SoGI gene expressed in high fiber and low fiber genotypes.

Figure 1A shows that, a total of 53,872 sequences out of 121,342 SoGI transcripts (~44%) were found to be expressed with an RPKM >0 in all samples studied, of which, 42,262 transcripts were commonly expressed in four groups of sample, comprising of top and bottom internodes of the high and low fiber groups. Of the total 51,792 transcripts expressed in the high fiber samples, there were 44,995 transcripts common between top and bottom internodes of high fiber, while 5,115 and 1,682 transcripts were unique to top internodes and bottom internodes of the high fiber samples, respectively. Of the total 50,879 transcripts expressed in the low fiber group, there were 44,490 transcripts common between the top tissues and bottom tissues of the low fiber group; 4,039 and 2,350 transcripts were unique to the top tissues and bottom tissues of the low fiber group, respectively. In general, if all transcripts with an RPKM >0 were considered as expressed transcripts, there were more transcripts expressed in the top tissues compared to the bottom tissues in the two groups of high and low fiber genotypes (3,433 and 1,689 more transcripts, respectively). When all transcripts expressed in the high and low fiber groups were compared, there were similar number of transcripts expressed in the two groups of high and low fiber genotypes (51,792 vs. 50,879), of which, 48,799 transcripts were common between the two groups, while 2,993 and 2,080 transcripts were unique to the high and low fiber groups, respectively (Fig. 1B).

### Transcripts differentially expressed during sugarcane stem maturation in high and low fiber genotypes

In total, 507 transcripts (Additional file 1) were found differentially expressed between the top and the bottom internodal samples of the high fiber group (termed as high-fiber T-B). Similarly, 160 transcripts (Additional file 2) were differentially expressed between the top and the bottom internodal samples of the low fiber group (termed as low-fiber T-B). For general function comparison, these DE transcripts, were annotated against the Gene Ontology (GO) database^29^, Mapman^30,31^ and KEGG (Kyoto encyclopaedia of Genes and Genomes) metabolic pathways^32,33^. BlastX analysis of the differentially expressed transcripts from the two experimental groups (high-fiber T-B and low-fiber T-B) showed “top hit species distribution” with *Sorghum bicolor*, *Setaria italica*, *Zea mays*, *Saccharum* hybrid cultivar R570 and cultivar ROC22 (Additional file 3: Fig. S1).

**Figure 2:**
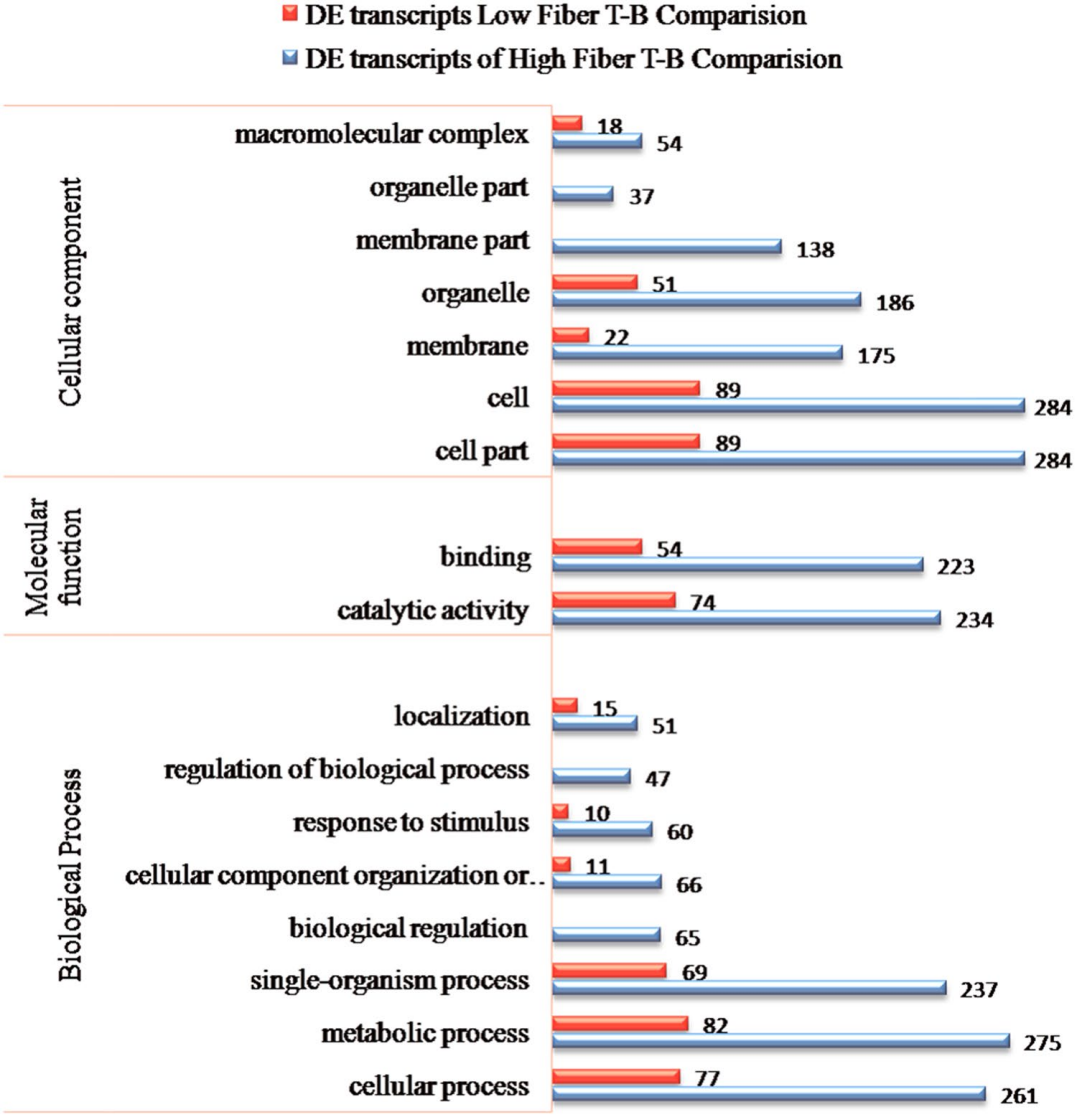
GO analysis for differentially expressed transcripts between top and bottom internodal tissues identified through BLAST2GO analysis.

Figure 2 presents GO analysis for the two sets of DE transcripts between top and bottom internodal tissues from high and low fiber groups, identified through BLAST2GO analysis^34^. The GO terms for the 507 DE transcripts were classified into three main classes, cellular component (CC), molecular function (MF) and biological process (BP). Among the cellular component category, the highest proportions of transcripts were involved in cell and cell part (284 transcripts, 56%), and organelle (186 transcripts, 36.7%). In molecular function, catalytic activity was prominent (234 transcripts, 46.2%), followed by binding (223 transcripts, 44%). In biological process, most of the transcripts were assigned to metabolic process (275 transcripts, 54.2%), cellular process (261 transcripts, 51.5%) and single organism process (237 transcripts, 46.7%). These GO terms for the 160 DE transcripts were grouped into BP towards metabolic process (82 transcripts, 51%), cellular process (77 transcripts, 48.1%) and single organism process (69 transcripts, 43.1%) and in CC towards cell and cell part (86 transcripts, 56%), and organelle (51 transcripts, 32%) and MF towards structural activity, molecular regulators and transporters (one transcript each, 0.63%), in addition to catalytic (74 transcript, 46.3%) and binding activity (54 transcripts, 33.8%) those found in the high-fiber T-B comparison. There were no transcripts for regulation of biological process/ biological regulation (in BP); and membrane part and organelle part (in CC).

MapMan analysis was employed to identify important functional groups of the total DE transcripts/genes activated in immature and mature tissues of sugarcane culm of the high and low fiber groups and visualize the transcript expression. In general, the Mapman annotation (Fig. S2 in Additional file 3) suggested that a large proportions of the DE transcripts were attributed to bins 29 (protein), 27 (RNA/transcription factors), 20 (stress), 34 (transport), 16 (secondary metabolism), 31 (cell), 33 (development), 26 (miscellaneous enzyme families), 21 (reduction-oxidation regulation), 30 (signaling), 10 (cell-wall) and 13 (amino acid metabolism).

**Figure 3:**
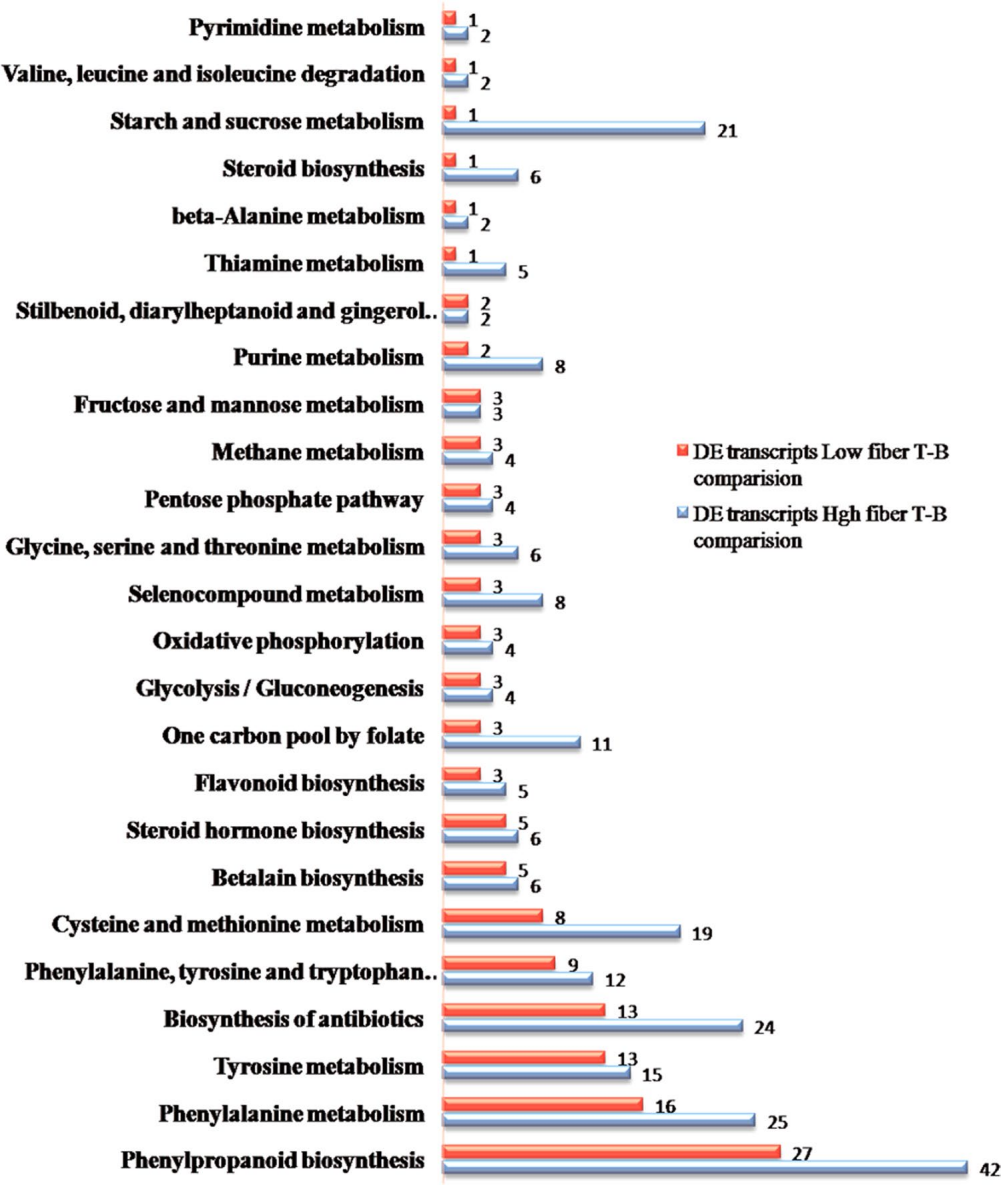
KEGG pathway analysis (http://www.kegg.jp/kegg/kegg1.html) for the differentially expressed transcripts of High Fiber Top-Bottom and Low fiber Top-Bottom comparisons.

KEGG metabolic pathway analysis provided additional information on possible function showing the pathways that the DE transcripts from the set of 507 transcripts and 160 transcripts identified in comparisons between top and bottom tissues for high and low fiber groups take part in. The results showed that the largest functional pathway was the phenylpropanoid biosynthesis (related to the synthesis of lignin monomers and flavonoid biosynthesis) followed by phenylalanine metabolic pathway, representing 8.28% and 4.9%, respectively, in the DE transcripts of the high-fiber T-B comparison. Biosynthesis of antibiotics, starch and sucrose metabolism, cysteine and methionine metabolism were also important pathways detected in the high fiber DE transcripts. Among the DE transcripts expressed in low-fiber T-B comparison the largest number of transcripts (16.8%) were related to the phenylpropanoid pathway (Fig. 3). Phenylalanine and tyrosine biosynthesis was also another important metabolic pathway in the low-fiber T-B comparison, to deliver the precursors for the phenylpropanoid pathway, followed by biosynthesis of antibiotics, cysteine and methionine metabolism pathways. There were other metabolic pathways (fructose and mannose metabolism, pyrimidine metabolism, arginine biosynthesis, beta-alanine metabolism, flavone and flavonol biosynthesis, galactose metabolism, nitrogen metabolism, pyruvate metabolism, steroid biosynthesis, valine, leucine and isoleucine degradation) differentially expressed in high-fiber T-B comparison transcripts with a small number of transcripts. The transcripts involved in starch and sucrose metabolism (carbohydrate metabolism) and the phenylpropanoid pathway were examined in detail to establish the enzymes that were expressed. We were able to identify a group of eleven enzymes from the high-fiber T-B comparison and a group of seven enzymes from the low-fiber T-B comparison that were involved in cellulose and lignin biosynthesis (Table 3), in which, we highlighted the enzymes that were only found in High-Fiber T-B comparison. These included cellulose synthase (CesA), gentiobiase, dehydrogenase and hydroxylase. KEGG maps for important pathways (starch and sucrose pathway and phenylpropanoid pathway) are shown in Figs S3 and S4 in Additional file 3, and the transcript IDs for every group of enzymes are provided in Tables S1 and S2 in Additional file 4. Functional details of the DE transcripts encoding enzymes in cellulose and lignin biosynthesis pathways will be discussed further in the next sections.

**Table 3:** Important enzymes encoded by the DE transcripts involved in starch and sucrose metabolism and phenylpropanoid pathway (http://www.kegg.jp/kegg/kegg1.html) of high-fiber top-bottom and low-fiber top-bottom comparisons (enzymes that were found only in high-fiber T-B are shown in bold).

High-Fiber T-B Comparison	Low-Fiber T-B Comparison
**EC 2**.**4**.**1**.**12 - cellulose synthase** (UDP-forming)	
**EC 3**.**2**.**1**.**21 - gentiobiase**	
EC 1.11.1.7 - lactoperoxidase	EC 1.11.1.7 - lactoperoxidase
**EC 1**.**1**.**1**.**195 - dehydrogenase**	
EC 4.3.1.24 - ammonia-lyase	EC 4.3.1.24 - ammonia-lyase
EC 4.3.1.25 - ammonia-lyase	EC 4.3.1.25 - ammonia-lyase
EC 6.2.1.12 - ligase	EC 6.2.1.12 - ligase
EC 1.2.1.44 - reductase	EC 1.2.1.44 - reductase
**E**.**C 1**.**14 - hydroxylase**	
EC 2.1.1.68 - O-methyltransferase	EC 2.1.1.68 - O-methyltransferase
EC 2.1.1.104 - O-methyltransferase	EC 2.1.1.104 - O-methyltransferase

### Differences between high fiber sugarcane and low fiber sugarcane at transcriptional level

To provide an insight into gene expression differences between the two groups of high and low fiber genotypes, DE transcripts identified from high-fiber T-B (507 transcripts) and low-fiber T-B (160 transcripts) were compared. A total of 134 transcripts were found to be common between the high-fiber T-B and low-fiber T-B groups, while a total of 373 and 26 transcripts were unique DE transcripts identified only in the high and low fiber genotypes, respectively. Mapman functional annotation of the three transcript sets is provided in Table 4.

**Table 4:** List of transcripts in each MapMan functional bins annotated for three sets of common and unique transcripts from high and low fiber genotypes.

Bin	Functional name	Number of DE transcripts		
		Common set	Unique to high fiber genotypes	Unique to low fiber genotypes
1	Photosynthesis	2	3	2
2	Major CHO metabolism	2	2	1
4	Glycolysis	—	2	—
6	Gluconeogenese/glyoxylate cycle	1	2	—
8	TCA/org. transformation	—	3	—
9	Mitochondrial electron transport/ATP synthesis	2	1	—
10	Cell-wall	—	18	—
11	Lipid metabolism	3	7	1
12	N-metabolism	—	1	—
13	Amino acid metabolism	19	31	5
15	Metal handling	5	7	2
16	Secondary metabolism	28	41	—
17	Hormone metabolism	2	12	1
18	Co-factor and vitamin metabolism	—	2	—
20	Stress	1	23	—
21	Redox.regulation	—	1	—
23	Nucleotide metabolism	—	3	—
25	C1-metabolism	2	3	2
26	Miscellaneous	5	24	1
27	RNA	5	23	—
28	DNA	—	4	—
29	Protein	5	29	1
30	Signaling	3	7	1
31	Cell	3	7	—
33	Development	1	7	—
34	Transport	1	21	1

In the common set of 134 DE transcripts between the high and low fiber genotypes, the most significant functional bins were assigned to “secondary metabolism” (28 transcripts), “amino acid metabolism” (19 transcripts), “metal handling”, “transcription factors/RNA”, “protein” and “miscellaneous” (5 transcripts each). Only a few transcripts (1–3 transcripts each) were annotated as “lipid metabolism”, “signalling”, “cell”, “photosynthesis”, “major carbohydrate metabolism”, “mitochondrial electron transport/ATP synthesis”, “hormone metabolism”, “C1-metabolism”, “gluconeogenese/glyoxylate cycle”, “stress”, development” and “transport”. These categories represented the common transcriptional functions differentially expressed that were detected in both high and low fiber genotypes in this study. Amongst these common DE transcripts, remarkable ones included those encoding several lignin biosynthesis genes (caffeic acid/5-hydroxyferulic acid O-methyl transferase – COMT, EC 2.1.1.68; cinnamoyl CoA reductase - CCR1, EC 1.2.1.44; caffeoyl CoA O-methyltransferase - CCoAOMT, EC 2.1.1.104; 4-coumarate CoA ligase - 4CL, EC 6.2.1.12 and phenylalanine ammonia lyase – PAL, EC 4.3.1.24), chalcone synthase 5 (CHS, EC 2.3.1.74), WLIM transcription factor (TF) homologs, MYB domain protein 61 (MYB61), MYB42, genes involving in synthesis/degradation of salicylic acid, synthesis/degradation of brassinosteroid, ATP-binding cassette transporter; guanine nucleotide-binding protein subunit beta-like protein (GPB-LR) (RWD); and IQ-domain 32 (IQD32) functioning in calmodulin binding.

Of the 373 DE transcripts in the high-fiber T-B comparison, 134 transcripts were up-regulated and 239 transcripts were down-regulated in the bottom internodal tissue. These transcripts represented DE genes that were detected in high fiber genotypes, but not in low fiber genotypes. It was also found that “secondary metabolism” and “amino acid metabolism” were the most predominant function bins with 41 and 31 transcripts annotated, respectively. A total of 12 to 29 DE transcripts were functionally assigned to bins “protein”, “miscellaneous”, “stress”, “transcription factors/RNA”, “transport”, “cell-wall” and “hormone metabolism”; while one to seven DE transcripts each were annotated as other MapMan bins. Notably, bin 16 “secondary metabolism” was composed of several transcripts from several pathways, including phenylpropanols/lignin and lignans (17 transcripts), dihydroflavonols (5 transcritps), flavonols (3 transcripts), shikimate (3 transcripts), glucosinolates (2 transcripts), one for each of terpenoids, wax, chalcones, anthocyanins, isoflovonoids and alkaloids-like pathways. Most of these transcripts were down-regulated in the mature tissues of the high fiber. It was revealed that enzymes involved in phenylpropanoid pathway including gentiobiase (EC 3.2.1.21), coumarate 3-hydroxylase (C3H, EC 1.14.14.1), ferulate 5-hydroxylase (F5H, EC 1.14.13) and cinnamyl-alcohol dehydrogenase (CAD, EC1.1.1.195) were identified as differentially expressed only in the high fiber genotypes. DE transcripts related to cellulose synthesis (in bin 10, cell-wall metabolism) specific to high fiber genotypes included 13 transcripts encoding CesAs (UDP-forming) (EC 2.4.1.12), COBRA-like 5 protein precursor (Protein BRITTLE CULM1), COBRA-like 6 protein precursor (BRITTLE CULM1-like 7 protein), COBRA-like 3 protein precursor (BRITTLE CULM1-like 4 protein) and two endo-1,4-beta-D-glucanase (endoglucanase 10, EC 3.2.1.4). Seven enzymes of starch and sucrose metabolism, including EC isomerase (EC 5.3.1.9), alpha-1,4 glucan phosphorylase L-2 isozyme (starch phosphorylase, EC 2.4.1.1) and sucrose phosphate synthase (EC 2.4.1.14, EC 2.4.1.13), were identified as differentially expressed only in the high fiber clones (Table S3 in Additional file 4). There were several DE transcripts relating to (a) TFs and RNA-binding proteins **(**bin 27, RNA/transcription factors) that were unique to high fiber genotypes, including those encoding PLATZ transcription factor family protein, WLIM1 transcription factor homolog, auxin-responsive protein IAA24, bZIP protein BZO2H2, basic leucine-zipper 44 (bZIP44), MYB86, CHY-type zinc finger family, C2H2 zinc finger family, C2C2(Zn) DOF zinc finger family and RNA transcription proteins; and (b) hormone metabolism (bin 17) especially DE transcripts were specific to high fiber including those involved in synthesis/degradation of salicylic acid, jasmonate, gibberellin, ethylene and brassinosteroid, and auxin induced regulated responses (NAD(P)-linked oxidoreductase superfamily protein and cytochrome b561/ferric reductase transmembrane). Additionally, there were transcripts involved in signaling including G-proteins (nucleolar GTP-binding protein, RAC-like GTP-binding protein 4 (GTPase protein ROP4), IQ-domain 32 (IDQ32) functioning in calmodulin binding, receptor kinases.leucine rich repeat II; and transport, including ligand-effect-modulator 3 (LEM3)-like, tonoplast intrinsic protein 1.1 (rTIP1) (plasma membrane intrinsic protein 1a) (PIP1a), efflux-type boron transporter, HCO_3_- transporter family, ATP-binding cassette transporter, major facilitator superfamily protein, nitrate transporter 1:2 (NRT1:2), PTR2 family proton/oligopeptide symporter, other peptide transporters and cation efflux family protein and phosphate translocator-like proteins.

Of the 26 DE transcripts unique in the low-fiber T-B comparison, 11 transcripts were down-regulated and 15 were up-regulated in the bottom internodal tissues respectively. The most predominant functional bins were “amino acid metabolism” containing five DE transcripts, while photosynthesis, “metal handling”, “C1-metabolism”, each had two DE transcripts assigned. “Major carbohydrates”, “lipid metabolism”, “hormone metabolism”, “protein”, “signalling” and “transport”, each had one transcript represented. Amongst these, sucrose synthase 1 (or SuSy, EC 2.4.1.13), abscisic acid - responsive protein-related, G-proteins (Pleckstrin homology domain superfamily protein) and sucrose transporter 2 (SUT2) were found only in low fiber genotypes (Additional file 2 and Table S4 in Additional file 4). SuSy, an important component of cellulose biosynthesis in the primary cell-wall^35^ as well as in the secondary cell-wall^36^, was found to be down-regulated in the bottom tissues of the low fiber genotypes. A non-specific lipid-transfer protein 3 precursor (LTP 3) predicted to encode a PR (pathogenesis-related) protein located in the cell-wall was also down-regulated in low fiber genotypes during maturation.

### DE transcripts specifically involved in cellulose and lignin deposition during sugarcane stem maturation in high and low fiber genotypes

**Figure 4:**
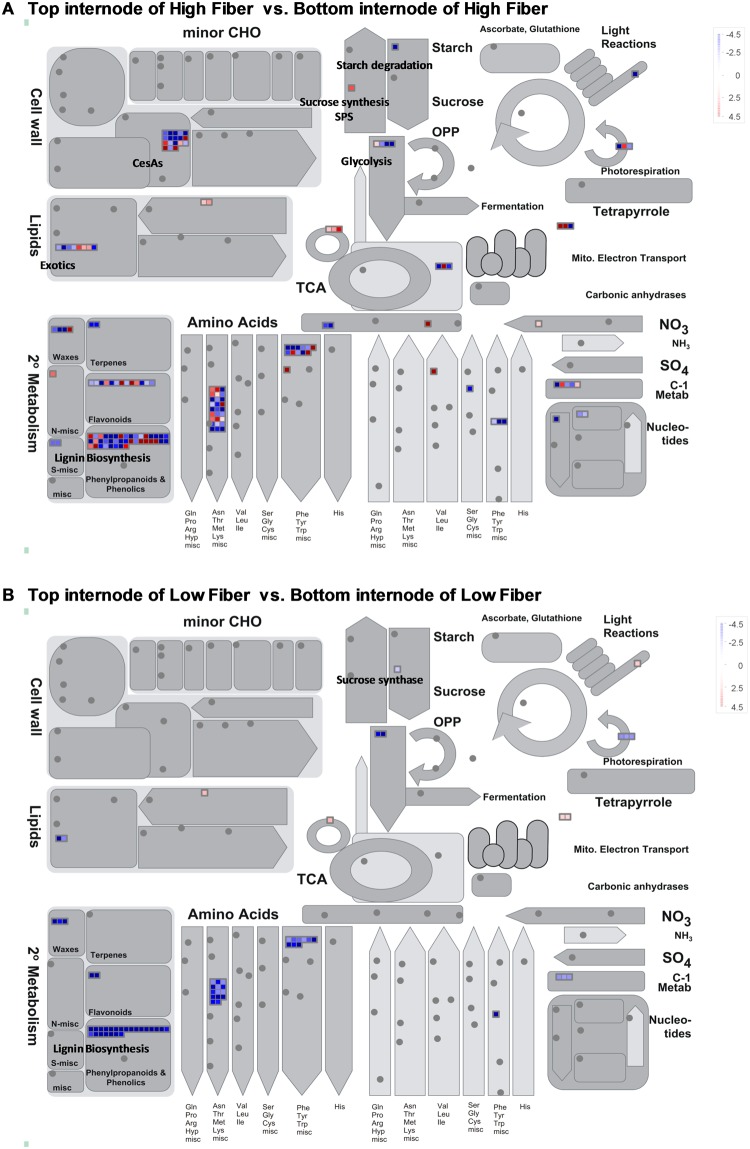
MapMan annotation of the differentially expressed transcripts, identified in high and low fiber genotypes. **(A**) Between top and bottom internodes of high fiber. (**B**) Between top and bottom internodes of Low fiber genotypes. Red color indicates up-regulation while blue color indicates down-regulation in the bottom internodal tissues.

The DE transcripts involved in cellulose and lignin deposition were further investigated. These CesA genes and lignin genes were categorized by MapMan mostly into two functional bins, bin 10 – cell-wall (including cellulose deposition) and bin 16 – secondary metabolism (including phenylpropanoid pathway) (Fig. 4A,B, Fig. S2 in Additional file 3). It is noteworthy that, there was a decrease in the expression of CesA with fold change (FC) from 1.89 to 2.9 and COBRA-like proteins (BRITTLE CULM1-like) with FC from 4.7 to 5.8 in the bottom internodes of high fiber groups. We were able to identify CesA2, CesA4 and CesA7 were down-regulated in the bottom internodes of the high fiber group and that these transcripts were not identified in low-fiber T-B comparison groups. The number of DE transcripts involved in cellulose synthase was identified for CesA2, CesA4; and 6 for CesA7. Taken this together with the results presented in the previous section, in terms on carbohydrate and sugar metabolism, this suggested that in the high fiber genotypes, the DE transcripts coding for CesA synthase (UDP-forming), COBRA-like proteins and endoglucanase 10 were down-regulated, whereas the transcripts coding for SPS (EC 2.4.1.14) and SuSy were down-regulated with FC of 2.81 in the bottom internodes of high fiber.

**Figure 5:**
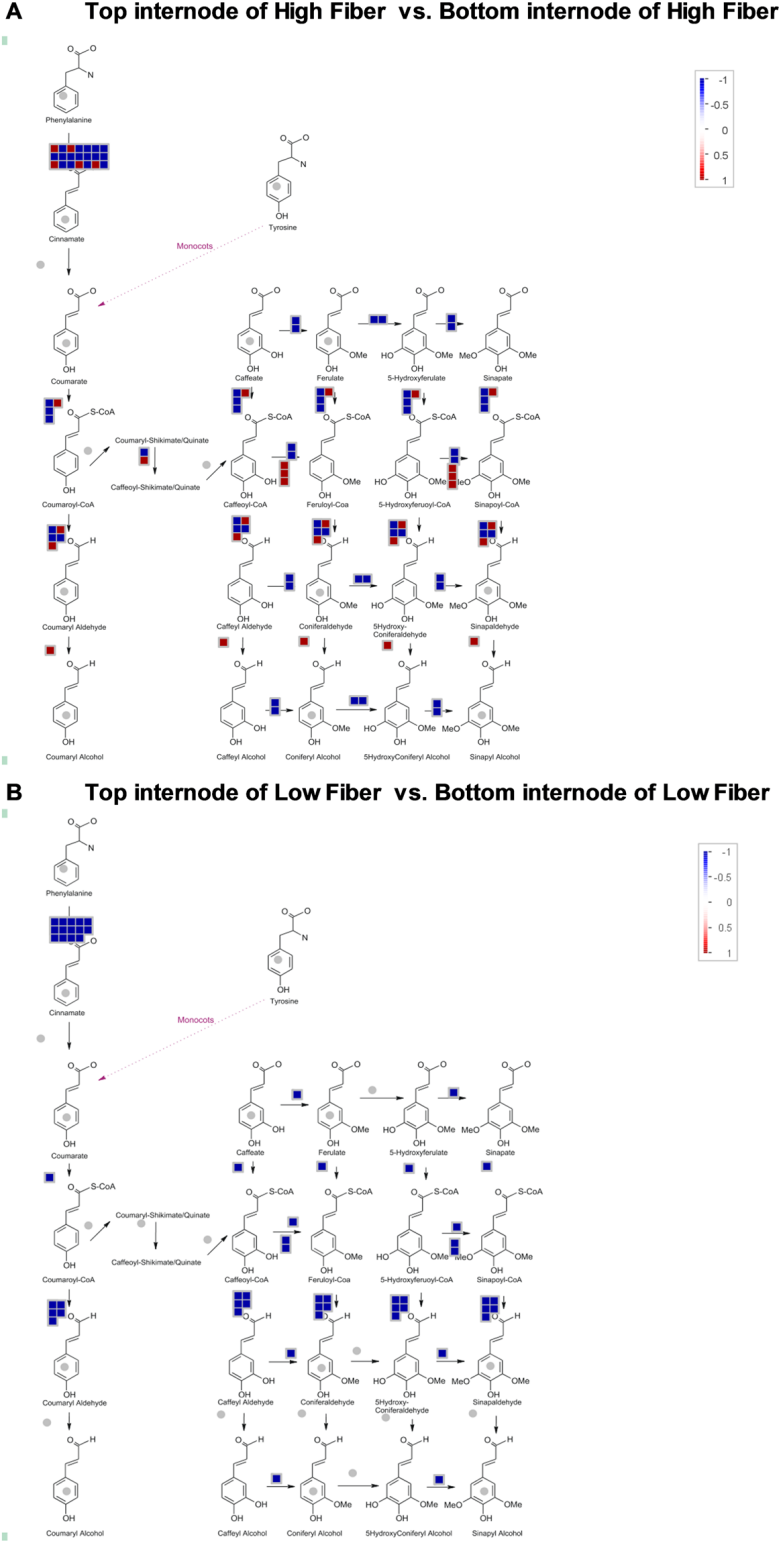
MapMan annotation of the differentially expressed transcripts involved in the phenylpropanoid pathway. (**A)** Between top and bottom internodes of high fiber. (**B**) Between top and bottom internodes of Low fiber genotypes. Red color indicates up-regulation while blue color indicates down-regulation in the bottom internodal tissues.

Several lignin related transcripts were revealed, including PAL, 4CL, C3H, CCoAOMT, F5H, CCR, COMT and CAD (see in Additional file 1). In total, 21 transcripts coding for PAL, four coding for 4CL, two for C3H, three coding for CCoAOMT, one coding for F5H, five coding for CCR, six coding for COMT, and one transcript coding for CAD, which are shown as blue boxes (down-regulated in the bottom internodes) in Fig. 5A,B. These transcripts were down-regulated during maturation in both high fiber and low fiber genotypes, and their fold changes were PAL (FC = −3.3 to −26.2), CCoAOMT (FC = −2.4 to −3.9), CCR (FC = −5 to −12), COMT (FC: −3.2 to −4.8) and 4CL (FC = −3.2 to −4.7). Among these, PAL was the most down-regulated transcripts with FC = 26.2. It is noteworthy that the fold change for PAL was higher in the top internodes of high fiber when compared to that of the low fiber genotypes indicating that the higher expression of entry point enzyme PAL, directs the carbon flux into the phenylpropanoid pathway and therefore, would be more likely to control the overall lignin content in the young tissues in sugarcane plant, especially in the high fiber genotypes. However, transcripts encoding C3H (FC = −2.4), F5H (FC = −2.6) and CAD (FC = −2.6) were found to be down-regulated exclusively in the high fiber genotypes. The exclusive expression of these transcripts indicates the sequential conversion of caffeoyl shikimic acid to syringyl lignin through coniferaldehyde and they are the major contributors for S/G modification in secondary cell-wall maturation.

### Transcription factors (TFs) involved in cellulose and lignin deposition during sugarcane stem maturation in high and low fiber genotypes

We investigated the TFs that potentially play important roles in regulating cellulose and lignin biosynthesis, as TFs are known for their transcriptional regulation roles in expression of cell-wall biosynthesis genes^37–39^. Previous studies showed that NAC, WRKY, bZIP, C2H2 ZF, homeobox, and HSF domain gene families were over-represented in those differentially expressed transcripts that were correlated with cellulose/lignin biosynthesis, and are thought to regulate secondary cell-wall development^40–42^. Co-expression network analysis of these TF encoding transcripts that related to cell-wall biosynthesis revealed that more TF transcripts were connected with monolignol biosynthesis, compared to that with cellulose and hemicellulose biosynthesis^42^. In our analysis, amongst the DE transcripts, we found that several of them encoded transcription factors (functional bin 27, Fig. S2 in Additional file 3) including LIM, MYB, auxin-responsive protein - IAA24, bZIP, zinc finger and PLATZ protein families.

WLIM1, MYB61 and MYB42 were found differentially expressed between the immature and mature tissues from both high and low fiber genotypes; while MYB86, PLATZ, IAA24, bZIP protein - BZO2H2, bZIP44, C2H2 zinc finger family, and C2C2 DOF zinc finger family were differentially expressed between immature and mature tissues in the high-fiber T-B comparison only. The LIM TF gene family was shown to be involved in key gene expression in the phenylpropanoid biosynthesis pathway, including PAL, hydroxycinnamate CoA ligase and CAD, and its expression was connected to lignin content^43^; while the MYB gene family (including MYB42, MYB61 and MYB86) has been shown to function as a master regulators for secondary cell-wall development and multiple monolignol biosynthesis genes including F5H, 4CL1 and HCT^39,44–51^. The C2C2 and C2H2 zinc finger families were also found to be related to secondary cell-wall biosynthesis^52^.

### Validation of DE genes by qPCR

**Figure 6:**
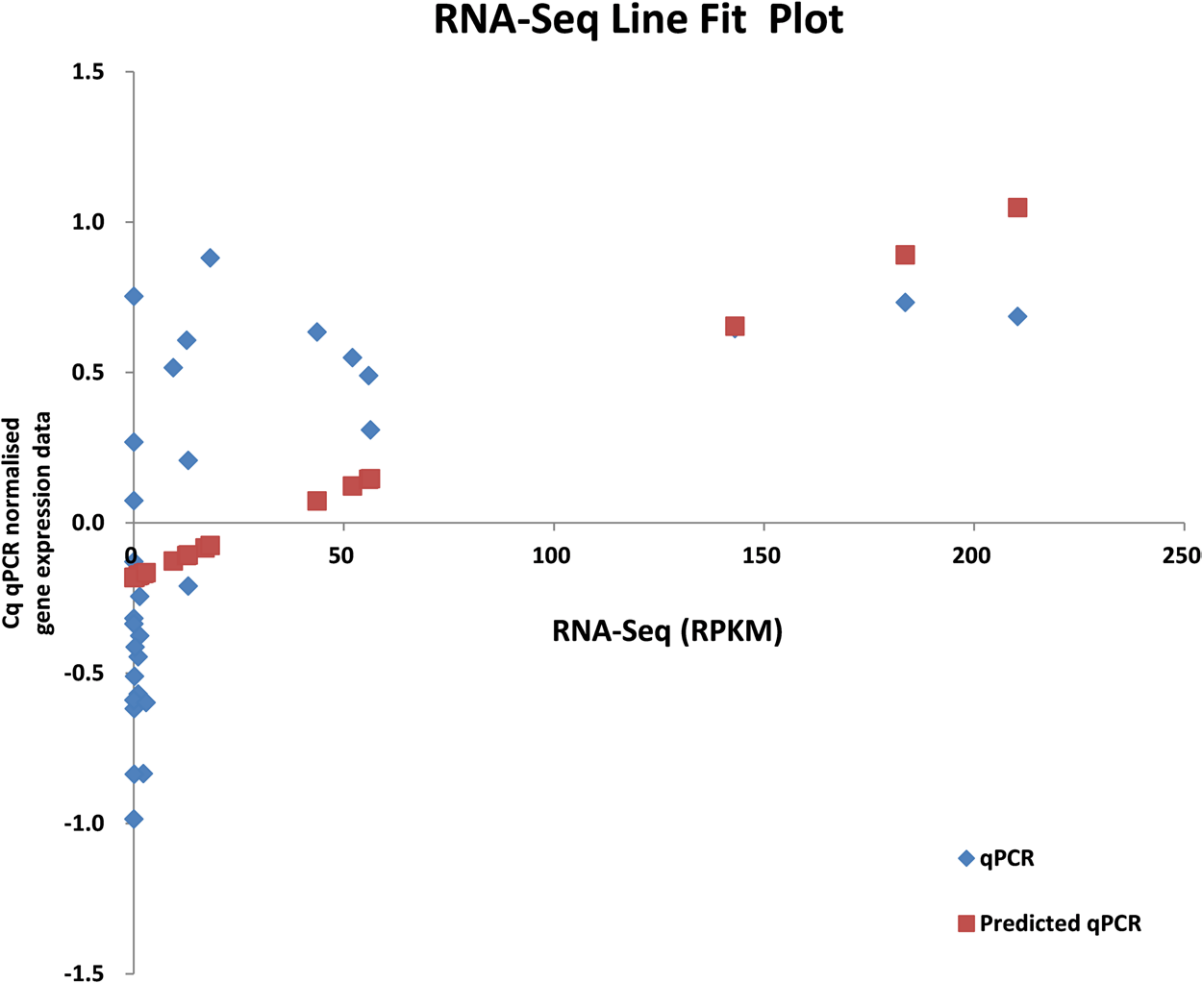
Correlation analysis of qPCR and RNA-Seq data of differentially expressed genes especially involved in lignin and cellulose biosynthesis of sugarcane.

The qPCR expression values previously reported^22^ same four identified DE genes, including CesA7, COBRA-like proteins (BC1l5), CCR and CAD (see Methods, for details), showed a significant correlation (*r* = 0.57, p < 0.001, n = 32, df = 30) with the RPKM values measured by RNA-Seq for a total of eight samples from four genotypes used this study. The correlation between the RNA-Seq and qPCR is shown as a fit line plot (Fig. 6). The detailed qPCR validation analysis is provided in Additional file 5.

## Discussion

This study was an effort to identify differences in the transcripts involved in the cellulose and lignin biosynthesis pathways in sugarcane varying in fiber content by conducting differential expression analysis between the young and mature internodal tissues from groups of high and low fiber genotypes. The comparison between young and the mature internodes could potentially underlie the transcripts/genes that are associated with carbon partitioning to the major biomass components (including cellulose and lignin) in the sugarcane culm over time in the two groups varying in fiber content.

Short-read data from Illumina platform was employed in RNA-Seq analysis in this study, since they provide sufficient depth and a lower error rate^53,54^. The use of the SoGI dataset^28^ as reference resulted in a low mapping percentage for every sample, ranging from 44 to 56%; while ~44% of sequences in the SoGI dataset had reads aligned. It could be that the SoGI database contains a proportion of short EST sequences (minimum 100 bp), while stringent mapping parameters were used in RNA-Seq analysis, which required at least 90% of the 150 bp-read length with 80% similarity to be mapped. Also, the SoGI was derived from 26 cDNA libraries of different tissues (leaf, stem and root), of different biotic and abiotic stressed plants^55,56^; while RNA-Seq reads in this study were generated only from the internodal tissues of the same condition; therefore it could have resulted in a low percentage of sequences in the reference dataset having reads mapped.

Global transcript expression analysis showed that the expression level of the two groups of high fiber and low fiber genotypes were very similar, with 48,799 transcripts found to be commonly expressed between the two groups, while only 2,993 and 2,080 transcripts were unique expressed in high and low fiber groups, respectively. A total of 533 differentially expressed transcripts were detected amongst high and low fiber genotypes in this study, of which, 134 DE transcripts were found common between the two groups, while 373 DE transcripts and 26 transcripts were unique to high fiber and low fiber genotypes. Functional analysis suggested that phenylpropanoid biosynthesis, phenylalanine metabolic pathway, biosynthesis of antibiotics, phenylalanine and tyrosine biosynthesis, biosynthesis of antibiotics, cysteine and methionine metabolism pathways were the most represented pathways in the differentially expressed transcripts in both high and low fiber genotypes. There was a significant number of transcripts that related to cell-wall metabolism (including several CesA genes, lignin genes and transcription factors) found in the DE transcript set that was unique to high fiber genotypes, while SUT2 and SuSy were found to be differentially expressed only in the low fiber genotypes. The DE transcripts that were involved in cell-wall biosynthesis, especially those encoding cellulose genes, lignin genes and transcription factors, were studied in detail.

Down-regulation of several CesA genes (UDP-forming) exclusively in the bottom internodes of the high fiber genotypes was in accordance with the results reported in an earlier study^57^. The down-regulation of CesA2, CesA4 and CesA7 in the bottom internodes of high fiber genotypes agreed with the findings in other studies^52,58^ that two major patterns of expression exist in sugarcane for CesA genes. Class I (ShCesA2, ShCesA6, ShCesA7, ShCesA9 and ShCesA12) showed high expression in the maturing stem only and class II (ShCesA3 and ShCesA5) showed high expression in both maturing and mature stems. Hence the transcripts identified for cellulose biosynthesis in this study can be grouped as class I transcripts expressed only in the maturing (top internodes) stem. Li, *et al*.^20^ classified CesA genes into two groups as primary CesA*s* (CesA involved in cellulose synthesis in primary cell-walls) and secondary CesA*s* (CesA*s* involved in cellulose synthesis in secondary cell-walls). However, a study by Zhong, *et al*.^59^ showed that a dominant mutant of CesA7 affected cell-wall formation in both types of walls indicating that CesA7 has structural properties allowing its incorporation into both primary and secondary cellulose synthase complexes. Earlier studies^60–62^ showed that deletion of CesA4, CesA7, or CesA8 resulted in a loss of rosette assembly thereby affecting the trafficking of CesA4 to cell-wall deposition sites in the secondary cell-wall. As we identified CesA2, CeSA4 and CesA7 to be differentially expressed only in the high-fiber T-B, we hypothesised that CesA4, or/and CeSA7 could be very useful for enhancing cellulose in the high fiber genotypes, which may be applicable for genetic engineering of cellulose biosynthesis in transgenic sugarcane in the future. The down-regulation of COBRA-like protein (protein BRITTLE CULM1) which plays an important role in anisotropic growth by orienting the deposition pattern of cellulose microfibrils could be associated with a lower level of cellulose crystallisation in the bottom internodes^63^. Analysis of lignin genes revealed gentiobiase, C3H, F5H and CAD were exclusively differentially expressed in high fiber genotypes. Gentiobiase is involved in hydrolysis of terminal, non-reducing beta-D-glucosyl 2-courmarinate to coumarinate with the release of coumarine^64^. The expression of gentiobiase (betaglucosidase, EC 3.2.1.21) in the top internodes of high fiber could result in enhanced content of coumarin^64^. According to Lattanzio^65^, coumarins are plant phenolics identified as internal physiological regulators or chemical messengers present in the insoluble or cell-wall fraction acting as reservoirs of phenylpropanoid units for lignin biosynthesis. The presence of coumarin also represents the beginnings of lignification of the secondary cell-wall. To our knowledge, this is the first report on the expression of gentiobiase in sugarcane and the role of the gene in the plant is not clear. The known cytochrome P450-dependent monooxygenases, C3H and F5H are considered to be an important hub controlling metabolic flux into G and S lignin monomers bythe ferulate production pathway^66^. The exclusive expression of the genes F5H and CAD drives the ρ-courmaryl coA to caffeoyl shikimic by the action of C3H, then to 5-hydroxy confialdehyde by F5H and finally to ‘S monomers of lignin by CAD; which was shown to gradually increase S monomer units as the stem matures in the top internodes of the high fiber group^67^. This trend in down-regulation of C3H, F5H and CAD in the bottom internodes of the high fiber group favours more syringyl units which confer rigidity, imperviousness and resistance to biodegradation to cell-walls that has been demonstrated in many reports^68^. During the early developmental stages, the culm acts as sink for sucrose, supporting cell-wall synthesis and cell expansion, without an increase in sucrose concentration^69^. Therefore, lignification starts in the early internode (top), and continues to the matured internode. In addition to genes, certain transcription factors like MYB86, PLATZ, IAA24, BZO2H2, bZIP44, C2H2 zinc finger family, and C2C2 DOF zinc finger family responsive for cellulose and lignin metabolism were also identified as the prominent players in carbon metabolism. According to previous reports^16,25,66^, fiber (cellulose, hemicellulose and lignin) in sugarcane is largely developmentally regulated and, consequently, it is expected that genes/ transcripts involved in biosynthesis are also developmentally regulated^70,71^. The sugarcane stem consists of a node and internodes, each at a different stage of development acropetally (with increasing maturity going down the stem). The cells in the immature internodes (top/younger internode) of the stem elongate initially, followed by the deposition of secondary cell-wall, suberisation and lignification^58,72^. The mature internodes (bottom/older internode) of a stem complete all growth and cell-wall thickening whilst the upper immature internodes are still growing^57^.

To conclude, in the current study we employed RNA-Seq and used the SoGI database to analyse the transcriptome of sugarcane top and bottom internode tissues from 20 genotypes varying in fiber content and identified candidate genes related to lignin biosynthesis. We identified a total of 507 and 160 transcripts differentially expressed between the top and bottom internodes of high fiber and low fiber genotypes, respectively. Functional analysis revealed that several CesA2, CesA4, CesA7, gentiobiase, C3H, F5H, CAD and several transcription factors were down-regulated only in the bottom internodes of the high fiber genotypes. CesA4 and CesA7 were identified as potential candidates for enhancement of cellulose in both (primary and secondary) cell-wall synthesis, while gentiobiase is a novel report for sugarcane and potentially plays a role in cell-wall biosynthesis. Further investigation of the role of gentiobiase in sugarcane is suggested. As expected, genes responsive to sucrose (sucrose synthase 1, and sucrose transporter 2) were found exclusively expressed in low fiber genotypes. These DE transcripts highlighted the difference in growth phase of the young and mature tissues and also those transcripts that were directly related to cell-wall metabolism. These results may assist in selection of potential genes/transcriptions factor for sugarcane biomass modification thereby reducing the cost of pre-treatment for biofuel and biomaterial production.

## Methods

### Plant material and data source

Twenty sugarcane genotypes were grouped into 10 high and 10 low fiber genotypes in such a way that each group consists of five commercial and five introgression lines (as shown in Table 1), provided by Sugar Research Australia. Genotype grouping was based on the fiber, hemicellulose, glucose and lignin percentage predicted through NIR described in^27^. The fourth internode from the top and the third internode from the bottom, hereby referred to as the “top intermodal tissue” and “bottom intermodal tissue” respectively, were harvested at 12^th^ months of age. Only the internodes were used and the nodes were discarded. Sample collection, RNA extraction, quality determination, cDNA library construction were described in^22,73^. In brief, 40 internodal samples comprising of 20 top and 20 bottom internodal tissues were collected and pulverized for RNA extraction, which followed protocol described in^74^. The quality, integrity and quantity of extracted RNA samples were assessed using a NanoDrop8000 spectrophotometer (ThermoFisher Scientific, Wilmington, DE, USA) for initial screen, and a 2100 Agilent Bioanalyser (Agilent Technologies, Santa Clara, CA, USA). Detail of Bioanalyser assessment for 40 RNA samples used in this study is provided in Additional file 6, which is adapted from^75^.

### Illumina sequencing and expression analysis

A total of 40 libraries, corresponding to the 20 top and 20 bottom internodal tissues respectively, were sequenced, in two lanes each as technical replicates, using an Illumina Hiseq4000 instrument at the Translational Research Institute, The University of Queensland, Australia. The paired-end raw reads were of 151 bp long obtained as fastq files which were imported into CLC genomics workbench ver. 9.0.4 (CLC-GWB, CLC Bio-Qiagen, Denmark) for further processing and analysis. The raw reads were initially trimmed to remove the adapter sequences (both universal and index adapters), then trimmed to remove the low quality reads (Phred quality score ≤Q20) and reads less than 35 bp in length. After pre-processing, the high-quality data (only the paired reads) obtained from each library from two technical replicates was combined and used for RNA-Seq analysis using *Saccharum officinarum* gene indices (SoGI) v3.0^28^ as a reference database that contains 121,342 contigs (SoGI statistics generated by^76^ are shown in Additional file 6).

RNA-seq data of the top and bottom internode tissue of the high and low fiber genotypes was compared in two combinations as follows: (1) top internodes were compared to the bottom internodes of the high fiber plants and (2) top internodes were compared to the bottom internodes of the low fiber plants. The default RNA-Seq parameters of 0.9 for “minimum length fraction”, of 0.8 for “minimum similarity fraction”, and maximum number of hits for a read of 10 were used in CLC-GWB. The expression levels were normalized in the RNA-seq analysis as RPKM (reads per kilobase of exon model per million reads)^77^. In global expression analysis, all SoGI transcripts with an RPKM >0 were counted as expressed and compared among top tissue, bottom tissue of the high fiber; top tissues, bottom tissue of the low fiber; and between high fiber and low fiber groups. Venn diagrams were created by a web-based tool InteractiVenn^78^. Statistically differentially expressed (DE) transcripts were identified using Empirical analysis of Differential Gene Expression (EDGE, a count based statistics) using expression values, with adjusted p-value using false discovery rate (FDR) corrected least significant difference set at the 0.05 level in CLC-GWB. The empirical analysis of DGE algorithm in the CLC-GWB is a re-implementation of the “Exact Test”, for two-group comparisons developed by Robinson and Smyth^79^. The original count data for a full expression experiment was used as input to the empirical analysis of DGE tool. The parameters were to estimate tag-wise dispersions and between “all pairs” of groups. In empirical analysis of DGE analysis, three columns were added to the experiment table for each pair of groups that are analyzed: the p-value, fold change and weighted difference. The fold change in gene expression was obtained and only those differentially expressed genes with an FDR adjusted p-value ≤ 0.05 between two groups were considered significant. In this analysis, the top internodal tissue samples were considered as the baseline (reference group) when compared to the bottom internodal tissue samples. If a transcript was up-regulated or down-regulated in the top tissues, it was equivalent to being down-regulated or up-regulated in the bottom tissues, respectively. Likewise, in the high and low fiber group comparison, the low fiber group was considered as the reference group.

### Functional annotation

The sequences of the differentially expressed transcripts (DE) identified in each experiment were extracted and subjected to BLASTX analysis against the NCBI non-redundant protein database (http://www.ncbi.nlm.nih.gov/) with an e-value threshold of 1e-10 and a maximum blast hits of 100 in CLC-GWB. Functional annotations for these four groups were conducted independently using BLAST2GO Pro ver 3.0.10^34^ with default parameters. All the DGEs were annotated, augmented using InterProScan and followed by “Run Annex” options and retaining the annotations pertaining to the plant database using the GO (gene ontology)-slim option in BLAST2GO. The DEs were also mapped against the KEGG (Kyoto encyclopaedia of Genes and Genomes) database^32,33^ containing metabolic pathways, which represent molecular interactions and reaction networks. Finally, pathway assignment was performed for all the transcripts on the basis of KEGG pathway maps using GO-enzyme code mapping option. The functional annotation was visualized in MapMan v3.5.1R2 program^30,31^ employing the mapping files of the DE transcripts generated by Mercator^30^.

### Validation of DE genes using quantitative real-time PCR (qPCR)

To validate the RNA-Seq differential expression, a correlation analysis was conducted using the expression values (RPKM) of four selected transcripts in this study and qPCR expression values (Cq qPCR normalised gene expression) of the same transcripts obtained from a separate study^22^. These selected transcript sequences were confirmed to be the same between the two databases, SoGI (used in this study) and SUGIT (used in^22^), by checking the primer sequences and amplicon length (detail is provided in Additional file 6). The RPKM values were obtained for top and bottom internodal tissue samples of four genotypes (QC02–402, Q200, QN05-803 and QBYN04-26041). The correlation analysis was performed in Microsoft Excel 2013.

### Data analysis

All CLC-GWB analyses and command-line driven Linus-based analyses were run on a QAAFI CLC Genomics Server and High Performance Computer clusters, respectively, provided by Research Computing Centre, The University of Queensland, Australia (https://rcc.uq.edu.au/). Other data analyses, unless otherwise stated, were performed in Microsoft Excel 2013.

### Availability of data and material

All RNA-Seq read data has been submitted as sequence read archive (SRA) in NCBI with the BioProject ID PRJNA356226, BioSample SAMN06323325, and accessions from SRR5258946 to SRR5259025 (80 accessions), as mentioned in^73^. Other relevant data are within the paper.

## Electronic supplementary material

Dataset 1

Dataset 2

Dataset 3

Dataset 4

Dataset 5

Dataset 6

## References

[1] Outlook, B. E. BP Energy outlook 2035. *bp*.*com/energyoutlook*, 53 (2015).

[2] Botha, F. Energy yield and cost in a sugarcane biomass system. *Proc*. *Aust*. *Soc*. *Sugarcane Tech*, 1–9 (2009).

[3] Lynd, L. R. *et al*. How biotech can transform biofuels. *Nat Biotech* **26**, 169–172, http://www.nature.com/nbt/journal/v26/n2/suppinfo/nbt0208-169_S1.html (2008). 10.1038/nbt0208-169 18259168

[4] Kajala K (2011). Strategies for engineering a two-celled C(4) photosynthetic pathway into rice. J Exp Bot.

[5] Zhu X-G, Long SP, Ort DR (2008). What is the maximum efficiency with which photosynthesis can convert solar energy into biomass?. Current Opinion in Biotechnology.

[6] Hoang NV, Furtado A, Botha FC, Simmons BA, Henry RJ (2015). Potential for Genetic Improvement of Sugarcane as a Source of Biomass for Biofuels. Front Bioeng Biotechnol.

[7] Kandel, R., Yang, X., Song, J. & Wang, J. Potentials, Challenges, and Genetic and Genomic Resources for Sugarcane Biomass Improvement. *Frontiers in plant science* **9**, 10.3389/fpls.2018.00151 (2018). 10.3389/fpls.2018.00151 PMC5821101 29503654

[8] de Souza AP, Grandis A, Leite DCC, Buckeridge MS (2014). Sugarcane as a Bioenergy Source: History, Performance, and Perspectives for Second-Generation Bioethanol. Bioenerg. Res..

[9] Luz SM, Gonçalves AR, Del’Arco AP (2007). Mechanical behavior and microstructural analysis of sugarcane bagasse fibers reinforced polypropylene composites. Composites Part A: Applied Science and Manufacturing.

[10] Canilha L (2012). Bioconversion of sugarcane biomass into ethanol: an overview about composition, pretreatment methods, detoxification of hydrolysates, enzymatic saccharification, and ethanol fermentation. Journal of biomedicine & biotechnology.

[11] Henry RJ (2010). Evaluation of plant biomass resources available for replacement of fossil oil. Plant biotechnology journal.

[12] Reddy MSS (2005). Targeted down-regulation of cytochrome P450 enzymes for forage quality improvement in alfalfa (Medicago sativa L.). Proceedings of the National Academy of Sciences of the United States of America.

[13] Chen F, Dixon RA (2007). Lignin modification improves fermentable sugar yields for biofuel production. Nature Biotechnology.

[14] Poovaiah CR (2014). Altered lignin biosynthesis using biotechnology to improve lignocellulosic biofuel feedstocks. Plant biotechnology journal.

[15] Aznar A (2018). Gene stacking of multiple traits for high yield of fermentable sugars in plant biomass. Biotechnology for biofuels.

[16] Bottcher A (2013). Lignification in sugarcane: biochemical characterization, gene discovery, and expression analysis in two genotypes contrasting for lignin content. Plant Physiol.

[17] Vanholme R (2012). Metabolic engineering of novel lignin in biomass crops. The New phytologist.

[18] Vicentini R (2015). Large-Scale Transcriptome Analysis of Two Sugarcane Genotypes Contrasting for Lignin Content. PLoS ONE.

[19] Santos Brito M (2015). Expression Profile of Sugarcane Transcription Factor Genes Involved in Lignin Biosynthesis. Tropical Plant Biology.

[20] Li S, Bashline L, Lei L, Gu Y (2014). Cellulose Synthesis and Its Regulation. The Arabidopsis Book /American Society of Plant Biologists.

[21] Demura T, Ye ZH (2010). Regulation of plant biomass production. Current opinion in plant biology.

[22] Hoang NV, Furtado A, O’Keeffe AJ, Botha FC, Henry RJ (2017). Association of gene expression with biomass content and composition in sugarcane. PLOS ONE.

[23] Cesarino I (2013). Expression of SofLAC, a new laccase in sugarcane, restores lignin content but not S:G ratio of Arabidopsis lac17 mutant. J Exp Bot.

[24] Papini-Terzi FS (2005). Transcription profiling of signal transduction-related genes in sugarcane tissues. DNA research: an international journal for rapid publication of reports on genes and genomes.

[25] Papini-Terzi, F. S. *et al*. Sugarcane genes associated with sucrose content. *Bmc Genomics* **10**, 10.1186/1471-2164-10-120 (2009). 10.1186/1471-2164-10-120 PMC2666766 19302712

[26] Casu R (2004). Identification of Differentially Expressed Transcripts from Maturing Stem of Sugarcane by in silico Analysis of Stem Expressed Sequence Tags and Gene Expression Profiling. Plant Mol Biol.

[27] Hoang NV (2016). High-Throughput Profiling of the Fiber and Sugar Composition of Sugarcane Biomass. Bioenerg. Res..

[28] SoGI. Saccharum officinarum Gene Indices. ftp://occams.dfci.harvard.edu/pub/bio/tgi/data/Saccharum_officinarum/. *Accessed on 20 June 2017* (2017).

[29] Ashburner M (2000). Gene ontology: tool for the unification of biology. The Gene Ontology Consortium. Nat Genet.

[30] Lohse M (2014). Mercator: a fast and simple web server for genome scale functional annotation of plant sequence data. Plant, cell & environment.

[31] Usadel B (2009). A guide to using MapMan to visualize and compare Omics data in plants: a case study in the crop species, Maize. Plant, cell & environment.

[32] Kanehisa M, Sato Y, Kawashima M, Furumichi M, Tanabe M (2016). KEGG as a reference resource for gene and protein annotation. Nucleic Acids Res.

[33] Kanehisa M, Goto S (2000). KEGG: kyoto encyclopedia of genes and genomes. Nucleic Acids Res.

[34] Conesa A, Gotz S (2008). Blast2GO: A comprehensive suite for functional analysis in plant genomics. International journal of plant genomics.

[35] Fujii S, Hayashi T, Mizuno K (2010). Sucrose synthase is an integral component of the cellulose synthesis machinery. Plant & cell physiology.

[36] P JC (2004). Genomics of cellulose biosynthesis in poplars. New Phytologist.

[37] Mitsuda N (2007). NAC transcription factors, NST1 and NST3, are key regulators of the formation of secondary walls in woody tissues of Arabidopsis. Plant Cell.

[38] Zhong R, Lee C, Zhou J, McCarthy RL, Ye ZH (2008). A battery of transcription factors involved in the regulation of secondary cell wall biosynthesis in Arabidopsis. Plant Cell.

[39] Zhong R, Richardson EA, Ye ZH (2007). The MYB46 transcription factor is a direct target of SND1 and regulates secondary wall biosynthesis in Arabidopsis. Plant Cell.

[40] Zhao Q (2010). Syringyl lignin biosynthesis is directly regulated by a secondary cell wall master switch. Proc Natl Acad Sci USA.

[41] Wang H (2010). Mutation of WRKY transcription factors initiates pith secondary wall formation and increases stem biomass in dicotyledonous plants. Proc Natl Acad Sci USA.

[42] Wang H (2016). Transcriptome analysis of secondary cell wall development in Medicago truncatula. BMC Genomics.

[43] Kawaoka A (2000). Functional analysis of tobacco LIM protein Ntlim1 involved in lignin biosynthesis. The Plant Journal.

[44] McCarthy RL, Zhong R, Ye ZH (2009). MYB83 is a direct target of SND1 and acts redundantly with MYB46 in the regulation of secondary cell wall biosynthesis in Arabidopsis. Plant & cell physiology.

[45] Liu J, Osbourn A, Ma P (2015). MYB Transcription Factors as Regulators of Phenylpropanoid Metabolism in Plants. Mol Plant.

[46] Kawaoka A, Nanto K, Ishii K, Ebinuma H (2006). Reduction of lignin content by suppression of expression of the LIM domain transcription factor in Eucalyptus camaldulensis. Silvae Genetica.

[47] Newman, L. J., Perazza, D. E., Juda, L. & Campbell, M. M. Involvement of the R2R3-MYB, AtMYB61, in the ectopic lignification and dark-photomorphogenic components of the det3 mutant phenotype. *Plant J* **37**, 10.1046/j.1365-313X.2003.01953.x (2004). 10.1046/j.1365-313x.2003.01953.x 14690508

[48] Goicoechea M (2005). EgMYB2, a new transcriptional activator from Eucalyptus xylem, regulates secondary cell wall formation and lignin biosynthesis. Plant J.

[49] Weng J-K, Li X, Bonawitz ND, Chapple C (2008). Emerging strategies of lignin engineering and degradation for cellulosic biofuel production. Current Opinion in Biotechnology.

[50] Taylor-Teeples M (2014). An Arabidopsis gene regulatory network for secondary cell wall synthesis. Nature.

[51] Sonbol FM (2009). The maize ZmMYB42 represses the phenylpropanoid pathway and affects the cell wall structure, composition and degradability in Arabidopsis thaliana. Plant Mol Biol.

[52] Hu R (2017). Transcriptome analysis of genes involved in secondary cell wall biosynthesis in developing internodes of Miscanthus lutarioriparius. Scientific reports.

[53] Chen J (2014). RNA-Seq for gene identification and transcript profiling of three Stevia rebaudiana genotypes. BMC Genomics.

[54] Dharshini S (2016). De novo sequencing and transcriptome analysis of a low temperature tolerant Saccharum spontaneum clone IND 00–1037. Journal of biotechnology.

[55] SUCEST-FUN Database. http://sucest-fun.org. *Accessed on 01 May 2015* (2015).

[56] Vettore AL, Silva FRD, Kemper EL, Arruda P (2001). The libraries that made SUCEST. Genetics and Molecular Biology.

[57] Casu RE, Jarmey JM, Bonnett GD, Manners JM (2007). Identification of transcripts associated with cell wall metabolism and development in the stem of sugarcane by Affymetrix GeneChip Sugarcane Genome Array expression profiling. Funct Integr Genomics.

[58] Jacobsen KR, Fisher D, Maretzki A, Moore P (1992). Developmental changes in the anatomy of the sugarcane stem in relation to phloem unloading and sucrose storage. Botanica Acta.

[59] Zhong R, Morrison WH, Freshour GD, Hahn MG, Ye ZH (2003). Expression of a mutant form of cellulose synthase AtCesA7 causes dominant negative effect on cellulose biosynthesis. Plant Physiol.

[60] Taylor NG, Laurie S, Turner SR (2000). Multiple cellulose synthase catalytic subunits are required for cellulose synthesis in Arabidopsis. Plant Cell.

[61] Taylor NG, Scheible WR, Cutler S, Somerville CR, Turner SR (1999). The irregular xylem3 locus of Arabidopsis encodes a cellulose synthase required for secondary cell wall synthesis. Plant Cell.

[62] Taylor NG, Howells RM, Huttly AK, Vickers K, Turner SR (2003). Interactions among three distinct CesA proteins essential for cellulose synthesis. Proceedings of the National Academy of Sciences of the United States of America.

[63] Roudier F (2005). COBRA, an Arabidopsis extracellular glycosyl-phosphatidyl inositol-anchored protein, specifically controls highly anisotropic expansion through its involvement in cellulose microfibril orientation. Plant Cell.

[64] Rahnamaie-Tajadod R, Loke KK, Goh HH, Noor NM (2017). Differential Gene Expression Analysis in Polygonum minus Leaf upon 24 h of Methyl Jasmonate Elicitation. Frontiers in plant science.

[65] Lattanzio V, Lattanzio VM, Cardinali A (2006). Role of phenolics in the resistance mechanisms of plants against fungal pathogens and insects. Phytochemistry: Advances in research.

[66] Bewg WP, Coleman HD (2017). Cell wall composition and lignin biosynthetic gene expression along a developmental gradient in an Australian sugarcane cultivar. PeerJ.

[67] Liu, Q., Luo, L. & Zheng, L. Lignins: Biosynthesis and Biological Functions in Plants. *International journal of molecular sciences* **19**, 10.3390/ijms19020335 (2018). 10.3390/ijms19020335 PMC5855557 29364145

[68] Yoon J, Choi H, An G (2015). Roles of lignin biosynthesis and regulatory genes in plant development. J Integr Plant Biol.

[69] Casu, R. E. *et al*. Tissue-specific transcriptome analysis within the maturing sugarcane stalk reveals spatial regulation in the expression of cellulose synthase and sucrose transporter gene families. *Plant Mol Biol*, 10.1007/s11103-015-0388-9 (2015). 10.1007/s11103-015-0388-9 26456093

[70] Menossi, M., Silva-Filho, M. C., Vincentz, M., Van-Sluys, M. A. & Souza, G. M. Sugarcane functional genomics: gene discovery for agronomic trait development. *International journal of plant genomics* **2008**, 10.1155/2008/458732 (2008). 10.1155/2008/458732 PMC2216073 18273390

[71] Mattiello L (2015). Physiological and transcriptional analyses of developmental stages along sugarcane leaf. BMC plant biology.

[72] Bull, T. *Manual of cane growing/edited by Mac Hogarth and Peter Allsopp*. (Bureau of Sugar Experiment Stations, 2000).

[73] Hoang NV (2017). A survey of the complex transcriptome from the highly polyploid sugarcane genome using full-length isoform sequencing and de novo assembly from short read sequencing. BMC Genomics.

[74] Furtado A (2014). RNA extraction from developing or mature wheat seeds. Methods in molecular biology.

[75] Hoang, N. V. *Analysis of genes controlling biomass traits in the genome of sugarcane (Saccharum spp*. *hybrids)* PhD Degree thesis, The University of Queensland, (2017).

[76] Gurevich A, Saveliev V, Vyahhi N, Tesler G (2013). QUAST: quality assessment tool for genome assemblies. Bioinformatics.

[77] Mortazavi, A., Williams, B. A., McCue, K., Schaeffer, L. & Wold, B. Mapping and quantifying mammalian transcriptomes by RNA-Seq. *Nat Meth* **5**, 621–628, http://www.nature.com/nmeth/journal/v5/n7/suppinfo/nmeth.1226_S1.html (2008). 10.1038/nmeth.1226 PMC13303166 18516045

[78] Heberle H, Meirelles GV, da Silva FR, Telles GP, Minghim R (2015). InteractiVenn: a web-based tool for the analysis of sets through Venn diagrams. BMC Bioinformatics.

[79] Robinson MD, Smyth GK (2008). Small-sample estimation of negative binomial dispersion, with applications to SAGE data. Biostatistics.

