# Supplementary material for: Transcriptome analysis highlights key differentially expressed genes involved in cellulose and lignin biosynthesis of sugarcane genotypes varying in fiber content
Source: Sci Rep. 2018 Aug 2;8:11612. doi: 10.1038/s41598-018-30033-4 (PMC6072797; doi:10.1038/s41598-018-30033-4)

Transcriptome analysis highlights key differentially expressed genes involved in cellulose and lignin biosynthesis of sugarcane genotypes varying in fiber content

Lakshmi Kasirajan[1,2,†], Nam V. Hoang[1,3,†], Agnelo Furtado[1], Frederik C. Botha[1,4] and Robert J. Henry[1]*

[1]Queensland Alliance for Agriculture and Food Innovation, The University of Queensland, St. Lucia, Queensland, Australia, 4072. [2]ICAR-Sugarcane Breeding Institute, Coimbatore 641007, Tamil Nadu, India. [3]College of Agriculture and Forestry, Hue University, Hue, Vietnam. [4]Sugar Research Australia, Indooroopilly, Queensland, Australia. 4068.

*Corresponding author: **Robert J. Henry**, Level 2, The John Hay Building, Queensland Biosciences Precinct [#80], 306 Carmody Road, The University of Queensland, St Lucia QLD 4072.

Email: robert.henry@uq.edu.au, Tel: +61 7 3346 0551, Fax: +61 7 334 60555

†These authors contributed equally to this work

Lakshmi Kasirajan

Email: K.Lakshmi@icar.gov.in,

Nam V. Hoang

Email: hoang.nam@uq.net.au

Agnelo Furtado

Email: a.furtado@uq.edu.au

Frederick C. Botha

Email: f.botha@uq.edu.au

Supplementary Figure 1. Blast top hit species distribution

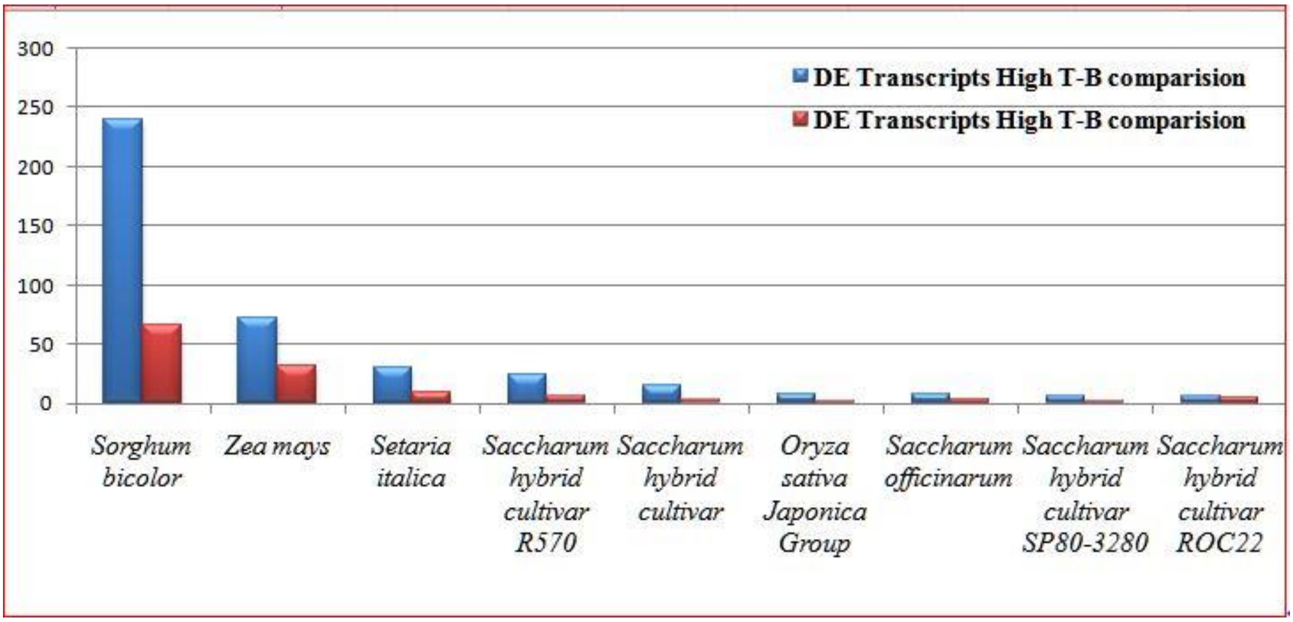

Supplementary Figure 2. Map man annotation bins

(a) DE transcripts between top and bottom internodes of high fiber

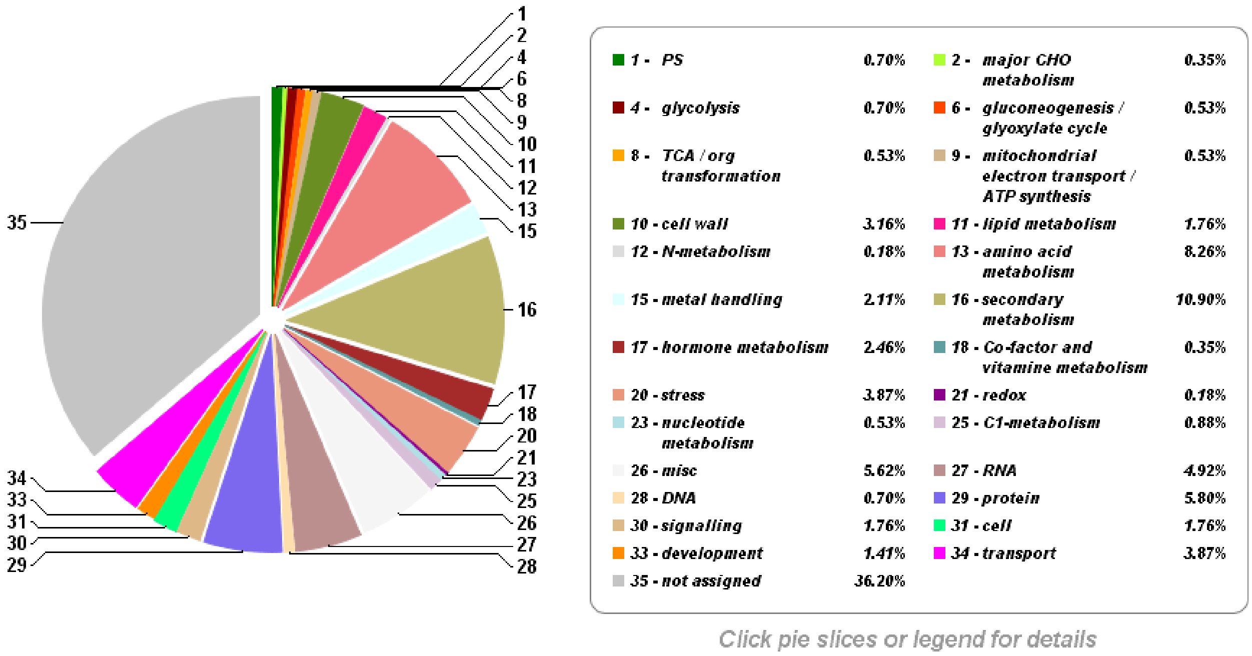

(b) DE transcripts between top and bottom internodes of Low fiber

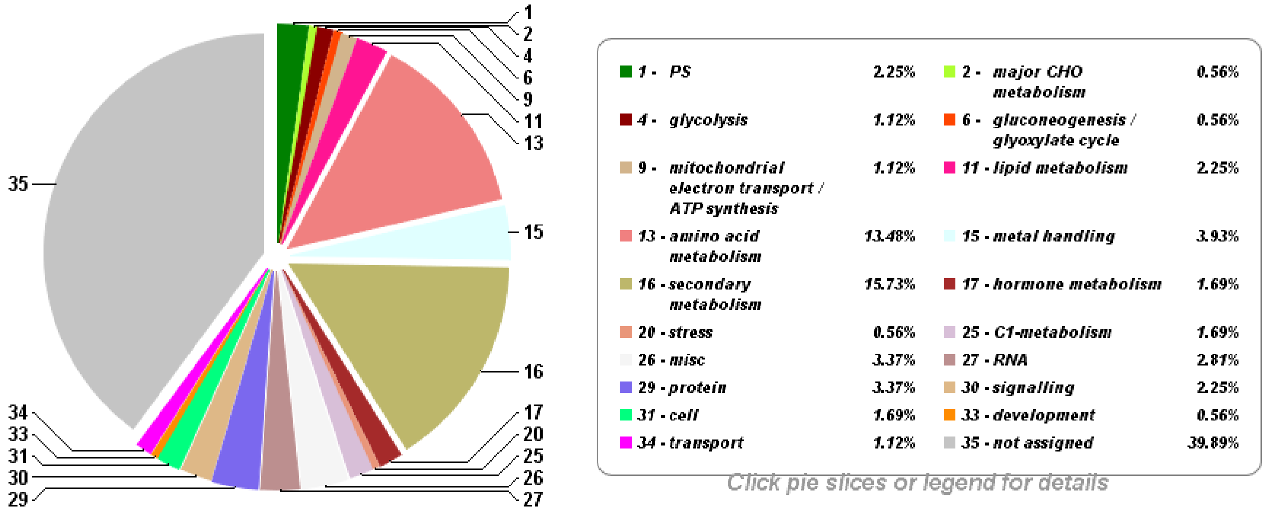

Supplementary Figure 3. KEGG maps of phenylpropanoid pathway (a) DE transcript between top and bottom internodes of High fiber, (b) DE transcript between top and bottom internodes of low fiber (http://www.kegg.jp/kegg/kegg1.html).

(a)

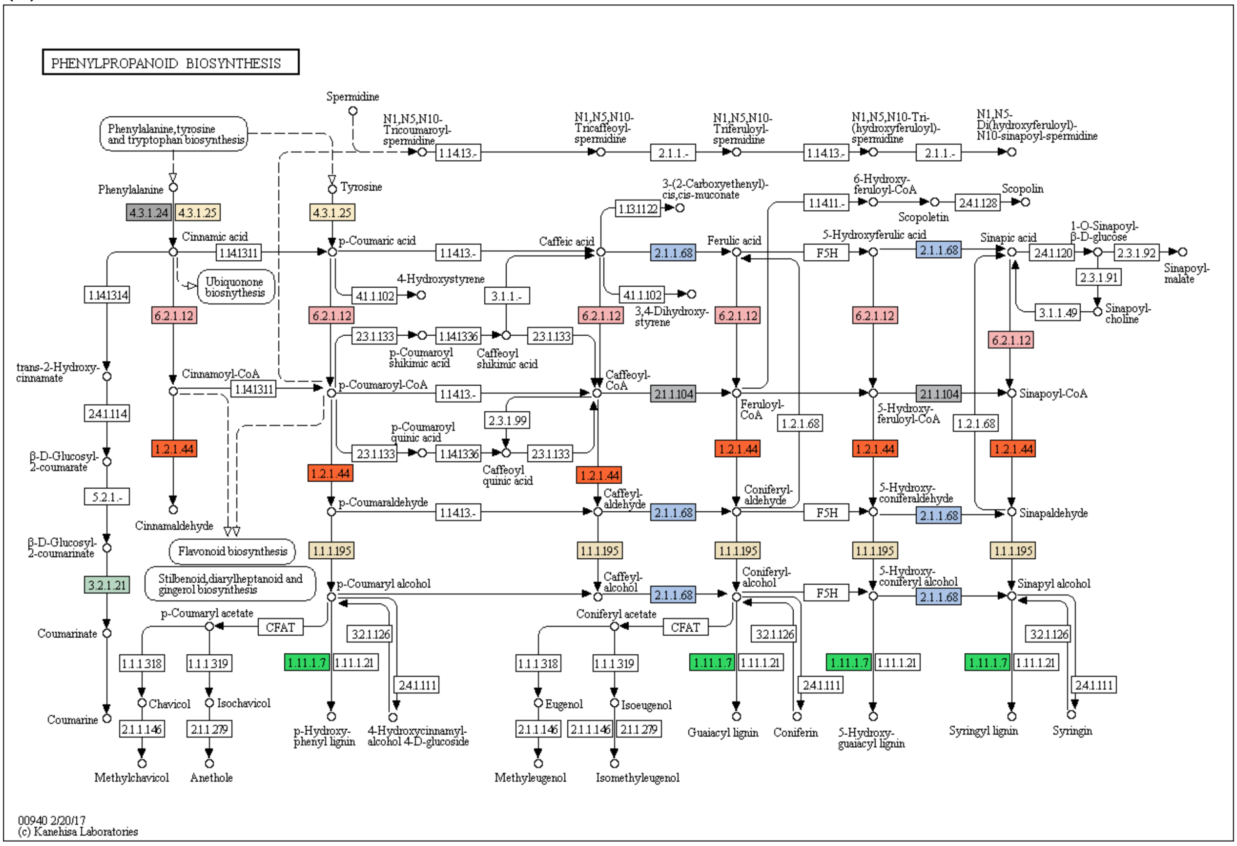

(b)

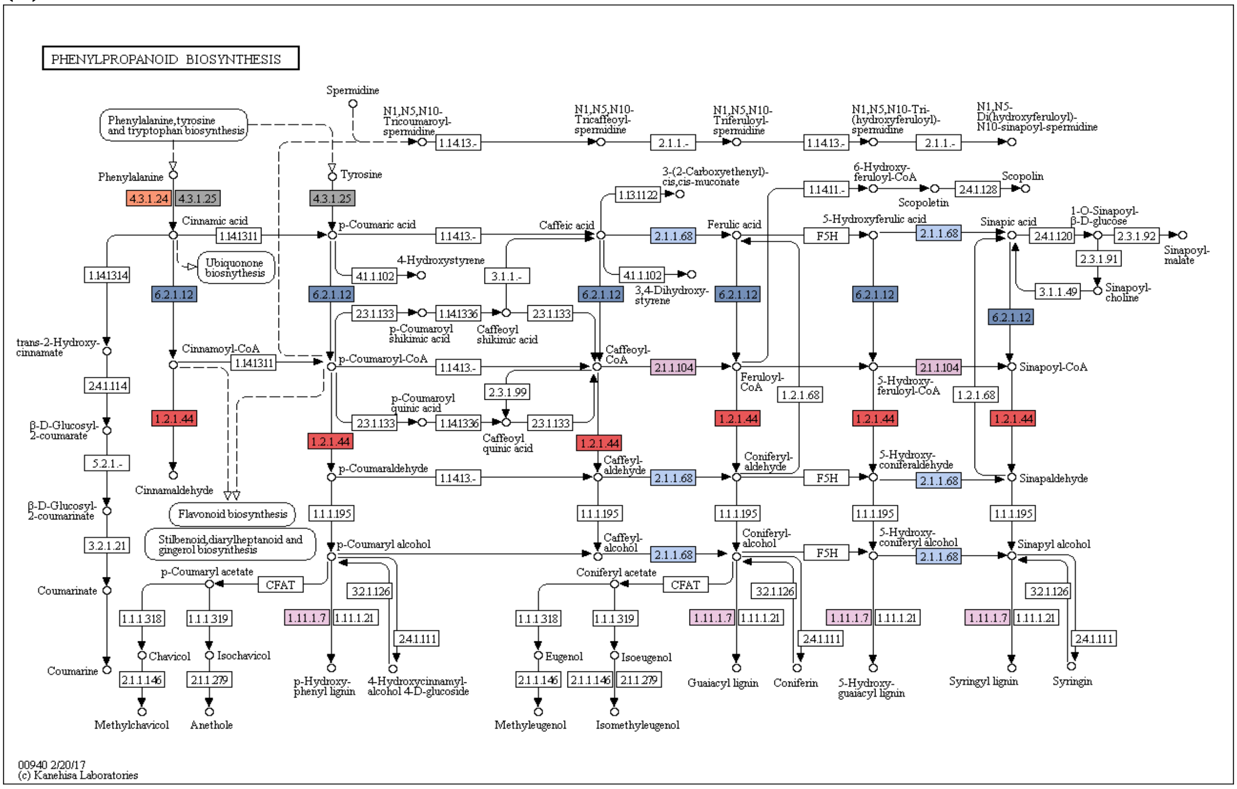

Supplementary Figure 4. KEGG maps of starch and sucrose pathway (a) DE transcript between top and bottom internodes of High fiber, (b) DE transcript between top and bottom internodes of Low fiber (http://www.kegg.jp/kegg/kegg1.html).

(a)

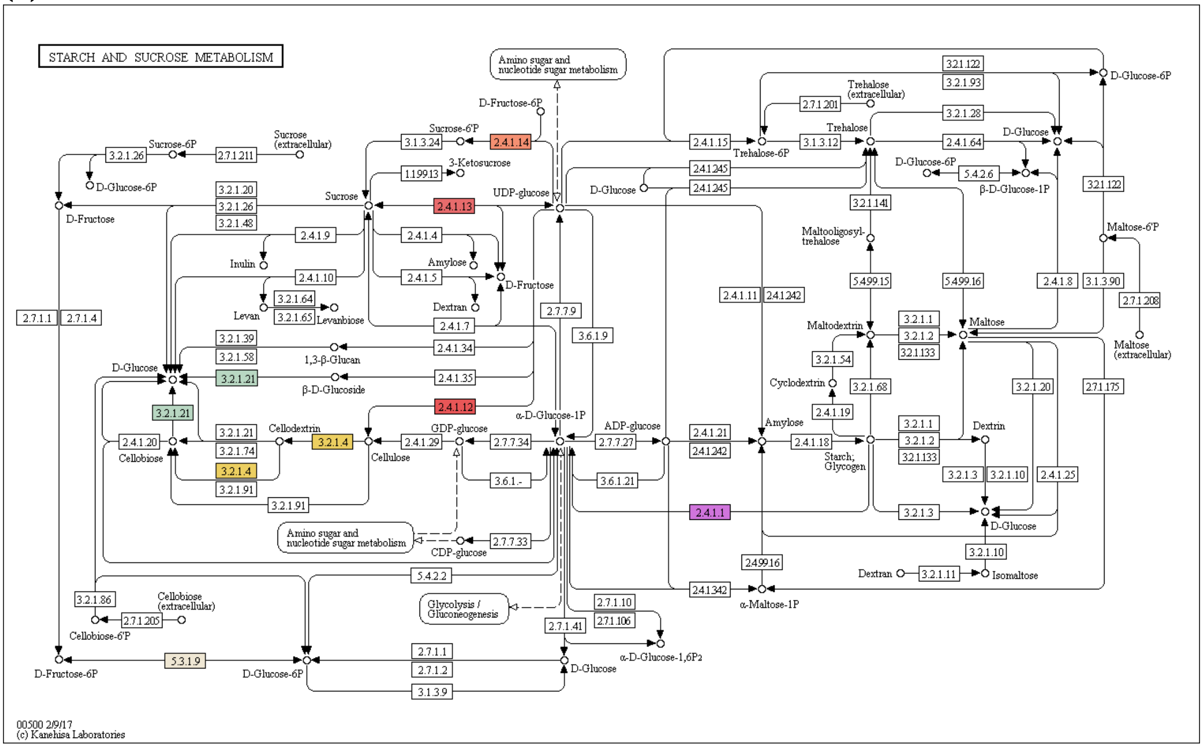

(b)

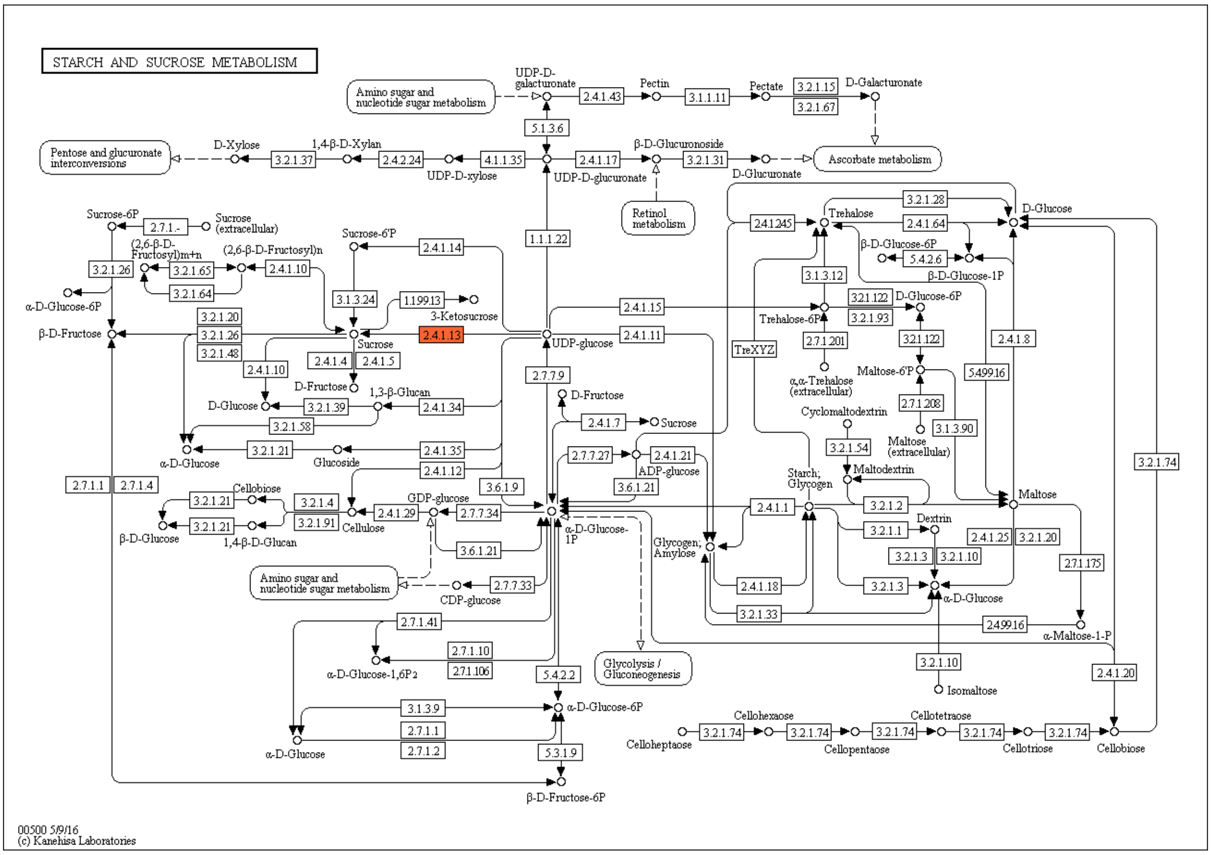

Supplement: Supplementary file 3 — Dataset 3 [file 41598_2018_30033_MOESM3_ESM.pdf]
