# Supplementary material for: Transcriptome analysis highlights key differentially expressed genes involved in cellulose and lignin biosynthesis of sugarcane genotypes varying in fiber content
Source: Sci Rep. 2018 Aug 2;8:11612. doi: 10.1038/s41598-018-30033-4 (PMC6072797; doi:10.1038/s41598-018-30033-4)
Supplement: Supplementary file 6 — Dataset 6 [file 41598_2018_30033_MOESM6_ESM.pdf]

# Transcriptome analysis highlights key differentially expressed genes involved in cellulose and lignin biosynthesis of sugarcane genotypes varying in fiber content

Lakshmi Kasirajan[1,2,†], Nam V. Hoang[1,3,†], Agnelo Furtado[1], Frederik C. Botha[1,4] and Robert J. Henry[1]*

[1]Queensland Alliance for Agriculture and Food Innovation, The University of Queensland, St. Lucia, Queensland, Australia, 4072. [2]ICAR-Sugarcane Breeding Institute, Coimbatore 641007, Tamil Nadu, India. [3]College of Agriculture and Forestry, Hue University, Hue, Vietnam. [4]Sugar Research Australia, Indooroopilly, Queensland, Australia. 4068.

*Corresponding author: **Robert J. Henry**, Level 2, The John Hay Building, Queensland Biosciences Precinct [#80], 306 Carmody Road, The University of Queensland, St Lucia QLD 4072. Email: robert.henry@uq.edu.au, Tel: +61 7 3346 0551,| Fax: +61 7 334 60555

†These authors contributed equally to this work

**Lakshmi Kasirajan**

Email: K.Lakshmi@icar.gov.in,

**Nam V. Hoang**

Email: hoang.nam@uq.net.au

**Agnelo Furtado**

Email: a.furtado@uq.edu.au

**Frederick C. Botha**

Email: f.botha@uq.edu.au

# ADDITIONAL METHODS

**Bioanalyser profiles of 40 internodal RNA samples used in this study.** Left panel: top internodal samples, right panel: bottom internodal samples (adapted from[1]).

**TOP INTERNODAL SAMPLES**

| | Sample | CODE | RIN | RNA Con. (ng/µl) | rRNA Ratio [25s / 18s] | Total (µg) in 50 µl |
|---|---|---|---|---|---|---|
| 1 | QC02-402 | T1 | 8.0 | 1776.3 | 1.8 | 88.8 |
| 2 | QA02-1009 | T2 | 8.1 | 505.4 | 1.9 | 25.3 |
| 3 | QN05-1460 | T3 | 8.2 | 788.0 | 2.0 | 39.4 |
| 4 | QN05-1743 | T4 | 8.0 | 678.5 | 1.9 | 33.9 |
| 5 | QN05-1509 | T5 | 8.1 | 575.2 | 1.8 | 28.8 |
| 6 | QS99-2014 | T6 | 8.0 | 1011.2 | 1.9 | 50.6 |
| 7 | QA96-1749 | T7 | 8.1 | 637.3 | 1.9 | 31.9 |
| 8 | Q241 | T8 | 8.2 | 1404.2 | 1.9 | 70.2 |
| 9 | Q200 | T9 | 8.0 | 1792.9 | 1.8 | 89.6 |
| 10 | QN05-803 | T10 | 8.0 | 2063.2 | 1.9 | 103.2 |
| 11 | KQB07-23863 | T11 | 7.8 | 1257.0 | 1.7 | 62.9 |
| 12 | KQB08-32953 | T12 | 8.2 | 1290.7 | 2.1 | 64.5 |
| 13 | KQB07-23990 | T13 | 8.1 | 618.6 | 2.0 | 30.9 |
| 14 | KQ08-2850 | T14 | 8.1 | 399.3 | 1.9 | 20.0 |
| 15 | KQB07-24619 | T15 | 8.5 | 963.7 | 1.9 | 48.2 |
| 16 | KQB07-24739 | T16 | 7.5 | 529.4 | 1.5 | 26.5 |
| 17 | QBYN04-26041 | T17 | 7.8 | 301.4 | 1.7 | 15.1 |
| 18 | KQB09-23137 | T18 | 7.8 | 392.1 | 1.8 | 19.6 |
| 19 | KQB09-20620 | T19 | 8.0 | 1008.9 | 1.9 | 50.4 |
| 20 | KQB09-20432 | T20 | 8.0 | 561.3 | 1.8 | 28.1 |

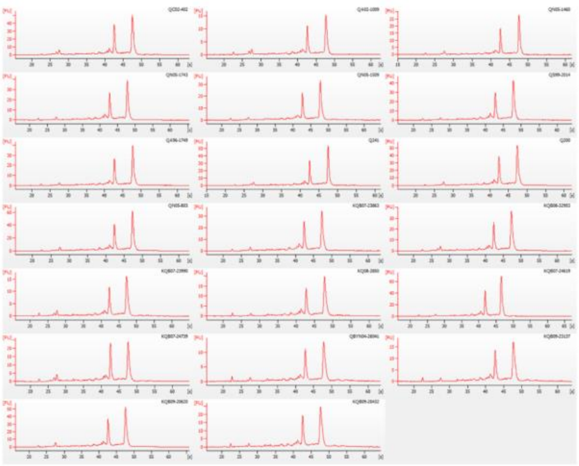

**BOTTOM INTERNODAL SAMPLES**

| | Sample | CODE | RIN | RNA Con. (ng/µl) | rRNA Ratio [25s / 18s] | Total (µg) in 50 µl |
|---|---|---|---|---|---|---|
| 1 | QC02-402 | B1 | 8.3 | 479.1 | 1.8 | 24.0 |
| 2 | QA02-1009 | B2 | 8.5 | 833.6 | 1.8 | 41.7 |
| 3 | QN05-1460 | B3 | 8.3 | 864.4 | 1.9 | 43.2 |
| 4 | QN05-1743 | B4 | 7.7 | 380.0 | 1.9 | 19.0 |
| 5 | QN05-1509 | B5 | 8.3 | 262.6 | 2.3 | 13.1 |
| 6 | QS99-2014 | B6 | 7.8 | 413.8 | 1.9 | 20.7 |
| 7 | QA96-1749 | B7 | 7.8 | 534.3 | 1.9 | 26.7 |
| 8 | Q241 | B8 | 8.6 | 1050.4 | 2.0 | 52.5 |
| 9 | Q200 | B9 | 8.2 | 1283.8 | 1.7 | 64.2 |
| 10 | QN05-803 | B10 | 8.1 | 684.6 | 2.2 | 34.2 |
| 11 | KQB07-23863 | B11 | 8.1 | 632.5 | 2.2 | 31.6 |
| 12 | KQB08-32953 | B12 | 8.2 | 770.7 | 2.0 | 38.5 |
| 13 | KQB07-23990 | B13 | 8.1 | 369.1 | 2.1 | 18.5 |
| 14 | KQ08-2850 | B14 | 8.0 | 442.8 | 2.0 | 22.1 |
| 15 | KQB07-24619 | B15 | 8.5 | 856.3 | 1.9 | 42.8 |
| 16 | KQB07-24739 | B16 | 8.4 | 865.5 | 1.9 | 43.3 |
| 17 | QBYN04-26041 | B17 | 7.9 | 714.0 | 2.2 | 35.7 |
| 18 | KQB09-23137 | B18 | 8.2 | 902.7 | 2.0 | 45.1 |
| 19 | KQB09-20620 | B19 | 8.6 | 539.2 | 2.1 | 27.0 |
| 20 | KQB09-20432 | B20 | 8.1 | 1021.9 | 2.0 | 51.1 |

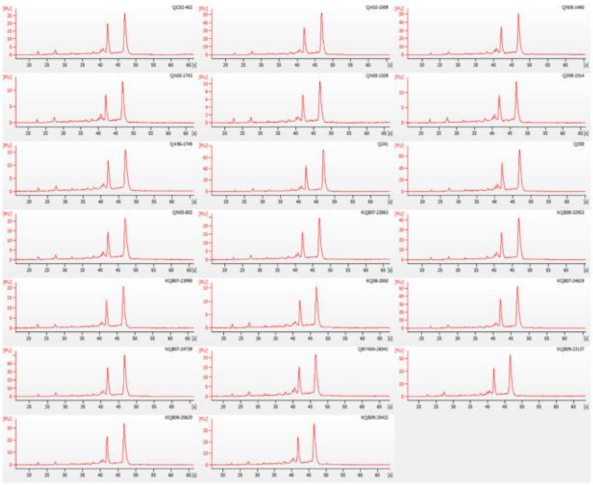

**Summary statistics of *Saccharum officinarum* gene index (SoGI) database**
(ftp://occamsdfciharvardedu/pub/bio/tgi/data/Saccharum_officinarum)

| **Statistics** | **SoGI** |
|---|---|
| Number of sequences (≥ 0 bp) | 121,342 |
| Number of sequences (≥ 1000 bp) | 13,279 |
| Number of sequences (≥ 5000 bp) | 0 |
| Total length (≥ 0 bp) | 88,397,709 |
| Total length (≥ 1000 bp) | 16,570,859 |
| Total sequences | 121,342 |
| Largest sequence | 4,854 |
| Total length | 88,397,709 |
| GC (%) | 52 |
| N50 | 729 |
| N75 | 641 |
| L50 | 46,463 |
| L75 | 78,851 |
| # N's | 125,596 |
| # N's per 100 kbp | 142 |

## qPCR primers and amplicon length from SoGI and SUGIT database

### 1. SoGI - TC113051 UniRef100_O82056 Cluster: Cinnamyl alcohol dehydrogenase; n=1; Saccharum officinarum

| | |
|---|---|
| N5_CAD_Forward | GTTCTTGCCGTTCTGTTCTGG |
| N5_CAD_R | CCACATCCTGGCGTACTTCG |

CGGCACGAGAGGCGCACTAGTTGTCGCGCTGCTTGCTTTCTTTCCGAAGGCTAAGCCACCCGGCCGGCTCGCTCGTCT
CCATCGCCCGCTCCCTCCGGGCCTCCGCCCCGCTCCGTCAGTCGTCTTCCCCGCGCCGCCGCCCGTAGCAGTCGATCGC
GGCCGGTCCCAACTCCCAGGAAGAGAATGGGGAGCCTGGCGTCCGAGAGGAAGGTGGTCGGGTGGGCCGCCAGGG
ACGCCACCGGACACCTCGCCCCCTACACCTACACCCTCAGGAGCACAGGCCCTGAAGATGTGGTGGTGAAGGTGCTCT
ACTGTGGGATCTGCCACACAGACATCCACCAGGCCAAGAACCACCTCGGGGCTTCAAAGTACCCCATGGTCCCTGGGC
ACGAGGTGGTCGGTGAGGTGGTGGAGGTCGGGCCCGAGGTGACCAAGTACGGCGTCGGCGACGTGGTAGGCGTCG
GGGTGATCGTCGGGTGCTGCCGCGAGTGCAACCCCTGCAAGGCCAACGTTGAGCAGTACTGCAACAAGAAGATCTGG
TCCTACAACGATGTCTACACTGACGGCCGGCCCACGCAGGGCGGCTTCGCCTCCACCATGGTCGTCGACCAGAAGTTT
GTGATGAAGATCCCGGCGGGTCTGGCGCCGGAGCAAGCGGCGCCGCTGCTGTGCGCGGGCGTGACGGTGTACAGCC
CGCTGAAAGCCTTTGGGCTGACGACCCCGGGCCTCCGTGGCGCGATCCTGGGCCTCGGCGGCGTGGGCCACATGGGC
GTGAAGGTGGCCAAGGCCATGGGCCACCACGTGACGGTGATCAGCTCGTCGTCCAAGAAGCGCGCGGAGGCGATGG
ACCACCTGGGCGCGGACGCGTACCTGGTGAGCTCGGACGCGGCGGCCATGGCGGCGGCCCCCGACTCGCTGGACTA
CATCATCGACACGGTGCCCGTGCACCACCCGCTGGAGCCCTACCTGGCGCTGCTGAAGCTGGACGGCAAGCACGTTCT
GCTGGGCGTCATCGGCGAGCCCCTCAGCTTCGTGTCCCCAATGGTGATGCTGGGGCGGAAGGCCATCACGGGGAGCT
TCATCGGCAGCATCGACGAGACCGCCGAGGTGCTCCAGTTCTGCGTCGACAAGGGGCTCACCTCCCAGATCGAGGTG
GTCAAGATGGGGTACGTGAACGAGGCGCTGGATCGGCTGGAGCGCAACGACGTCCGCTACCGCTTCGTCGTCGACGT
CGCCGGCAGCAACGTCGAGGAGGTGGCGGCCGATGCGCCGAGCAACTGACGTCGGCGCTGCGTCGTCGAACCGTGA
GGGGAGGTTGTGCCCCGCGTGCAACGTTCGTTCGTGTCGAGGCTGCGTGCGCTTCTGCTTCCTTTAGTAGTTGTGGGC
TTGTCGC**GTTCTTGCCGTTCTGTTCTGG**GCTTTCAGATGAGCTGATGGATGGTCTGTTTAAATGTCATCAGACTAAATA
ACTATGTGTAGTACTGTAGTACGTTTAATACT**CGAAGTACGCCAGGATGTGG**TGTGGTGTCACCAGCAATCTGGATTT
GCCCAAGTGTGAAGTGTTTCTCTTTTTTCTTC

**Amplicon = 124 bp**

### SUGIT Cinnamyl alcohol dehydrogenase

ACCCTACTCACCCAAAAAGACAGGCGCACTAATTGTCGCGCTGCTTGCTTTCTTTCCGAAGGCTAAGCCACCCGGCCG
GCTCGCTCGTCTCCATCGCCCGCTCCCTCCGGGCCTCCGCCCCGCTCCGTCGGTCGTCTTCCCCGCGCCGCCGCCCGTA
GCAGTCGATCGCGGCCGGTCCCAACTCCCAGGAAGAGAATGGGGAGCCTGGCGTCCGAGAGGAAGGTGGTCGGGT
GGGCCGCCAGGGACGCCACCGGACACCTCGCCCCCTACACCTACACCCTCAGGAGCACAGGCCCTGAAGATGTGGTG
GTGAAGGTGCTCTACTGTGGGATCTGCCACACAGACATCCACCAGGCCAAGAACCACCTCGGGGCTTCAAAGTACCCC
ATGGTCCCTGGGCACGAGGTGGTCGGTGAGGTGGTGGAGGTCGGGCCCGAGGTGACCAAGTACGGCGTCGGCGAC
GTGGTAGGCGTCGGGGTGATCGTCGGGTGCTGCCGCGAGTGCAACCCCTGCAAGGCCAACGTTGAGCAGTACTGCA
ACAAGAAGATCTGGTCCTACAACGATGTCTACACTGACGGCCGGCCCACGCAGGGCGGCTTCGCCTCCACCATGGTCG
TCGACCAGAAGTTTGTGATGAAGATCCCGGCGGGTCTGGCGCCGGAGCAAGCGGCGCCGCTGCTGTGCGCGGGCGT
GACGGTGTACAGCCCGCTGAAAGCCTTTGGGCTGACGACCCCGGCCCTCCGTGGCGCGATCCTGGGCCTCGGCGGCG
TGGGCCACATGGGCGTGAAGGTGGCCAAGGCCATGGGCCACCACGTGACGGTGATCAGCTCGTCGTCCAAGAAGCG
CGCGGAGGCGATGGACCACCTGGGCGCGGACGCGTACCTGGTGAGCTCGGACGCGGCGGCCATGGCGGCGGCCGC

CGACTCGCTGGACTACATCATCGACACGGTGCCCGTGCACCACCCGCTGGAGCCCTACCTGGCGCTGCTGAAGCTGGA
CGGCAAGCACGTTCTGCTGGGCGTCATCGGCGAGCCCCTCAGCTTCGTGTCCCCAATGGTGATGCTGGGGCGGAAGG
CCATCACGGGGAGCTTCATCGGCAGCATCGACGAGACCGCCGAGGTGCTCCAGTTCTGCGTCGACAAGGGGCTCACC
TCCCAGATCGAGGTGGTCAAGATGGGGTACGTGAACGAGGCGCTGGATCGGCTGGAGCGCAACGACGTCCGCTACC
GCTTCGTCGTCGACGTCGCCGGCAGCAACGTCGAGGAGGTGGCGGCCGATGCGCCGAGCAACTGACGTCGGCGCTG
CGTCGTCGAACCGTGAGGGGAGGTTGTGCCCCGCGTGCAACGTTCGTTCGTGTCGAGGCTGCGTGCGCTTCTGCTTCC
TTTAGTAGTTGTGGGCTTGTCGC**GTTCTTGCCGTTCTGTTCTGG**GCTTTCAGATGAGCTGATGGATGGTCTGTTTAAAT
GTCATCAGACTGAATAACTATGTGTAGTACTGTAGTACGTTTTATACT**CGAAGTACGCCAGGATGTGG**TGTGGTGTCA
CCAGCAATCTGGATTTTTTTTATAGAACATACCTGCATTATAGTTAAGG

**Amplicon = 124 bp**

## 2. SoGI - CA165461 homologue to UniRef100_O82055 Cluster: Cinnamoyl-CoA reductase; n=1; Saccharum officinarum

| | |
|---|---|
| N7_CCR_Forward | GTAGTAGTGCCGAAGCAGAAAG |
| N7_CCR_Reverse | CCTAGCTACCAACTCGGTCATC |

CGATCGCAAGAGAGAATCCTGCCAAA**CCTAGCTACCAACTCGGTCATC**ACATCACTCGACCGCACACACAAAGGGGA
ACCTAAAAACTAACTCTCTTCAACTTCCTCTGC**CTTTCTGCTTCGGCACTACTGC**TACGAGACACACCGCGAGCTAGCTA
GCTTTCGTGACGAACTAGCTAAAGAGGAACAGCTGGTCGGTCTCTTATCATCATCGCCGCGCCAAGATGACCGTCGTC
GACGCCGTGTCCACTGATGCCGCCGGCGCCCCTGCAGCCGCCGCGGCACCGGTGCAGCAGCCCGGGAACGGGCAGA
CCGTGTGCGTCACCGGCGCGGCCGGGTACATCGCCTCGTGGCTGGTCAAGCTGCTGCTCGAGAAGGGATACACTGTC
AAGGGAACCGTCAGGAACCCAGATGACCCGAAGAACGCGCACCTCAAGGCGCTGGACGGCGCCGCCGAGCGGCTGA
TCCTCTGCAACGCCGACCTCCTGCACTACGACGCCATCTGGCGCCCCGTGCAGGCGTGCCATGGCGTTCTTCCACACCG
CCCCCCCGGTCACCGACGACCCCCGATCAAAATGGTTGAACCCTGCCGGTCGCGCGGGCTACGACATAACTCTATCCA
CCCGCGAGGCGGAAGCCCGGAACCCGTGCAGCGAGGTGGTGTTTCACCTCCTCCC

**Amplicon = 106 bp**

### SUGIT Cinnamoyl-CoA reductase

TTTTTTTTTTTTTTTTTTTTTTTCTAGAAATAAATGAACATTAGCCATTTTTATTCACTCATCACTATAGTCCACAAGAACGA
ACATGACGTTACAACAAGCTTAGGGTAGGGGTATCAGCCACCGAACAAACACTAGTTTGTTTACACAGCTGGGGCAG
GGCTGTATGAACATTGCTTTTCTGTTCATGTGTACGTGGCATCTCTCTCTCACGCACGGATGGCGATTCCTCCCTGCTGC
AGCTCGGCGGCGGCGGGGGCCGCCTCCTTGTCGTCGGCCTCCGTCGTCTGCTCTCCGAGCACCGGCAGGTGGCCCTTC
TCCTGAAGGTTCTTCACCGTGTCGTACAGCGACTGGCTCACCGGCCGGAACTCCAGCCCCAGGTCCCGGAGCTTCTGG
TTCGAGAACTTGTACGGCTGCTTCCGCGGGTTCACCTCGTCGGAGCACCTGGTGGGGACGGGGTACTCGGGGAAGAG
CTTGGCGAGGATGCGGACGACGTCCTCGCGGTGGAGGACGCGCTCGGCGCAGAGGTATCGGCCGGACGCGCGCGG
GCTCTCGAAGACGCGGAGGTGCGCGTCGGCGACGTCGCGGACGTCCACGTACGCCTGCACGGCGTTGGCGAAGGTG
CGCGCGGAGCCGTCCAGGTACTTGGCCACGTGCGCGATGCTGGCGTTCACCGTCGGCTGCAGCAGTGGGCCCACCAC
CAGCACCGGGTTCACCACCACCAGGTCCACGCCGCGCTGCCGGGCCGCGTCCCACGCCGCCTGCTCCGCCACCGCCTT
GCCGTAGCAGTACCAGTTCCTGGTTTTCTTGCAGAACTCGAGGTCGCTCCAGCACGACTCGTCGACCACGACGTCGGG
CCCGCGGCTGGGGTCCATGGTCACCGCGCCGATGGAGGACGTGAACACCACCCGCCGCACCGTGCCGGCCTCCGCCG
CCGCGTTGATCACGTACTCCGTGCCGCGCACCGCCGGCTCCACCATTTGCTCCGGGTCGTCGGTGACCGGGGAGGCG
GTGTGGAAGACGCCATGGCAGCCCTGCACGGCGCGGCAGATGGCGTCGTAGTCAAGGAGGTCGGCCTTGCAGAGGA

TCAGCCGCTCGGCGGCGCCGTCCAGCGCCTTGAGGTGCGCGTTCTTCGGGTCATCTGGGTTCCTGACGGTTCCCTTGA
CAGTGTATCCCTTCTCGAGCAGCAGCTTGACGAGCCACGAGGCGATGTACCCGGCCGCGCCGGTGACACACACAGTC
TGCCCGTTCCCCGCGGGCTGCACAGGCGCCGCGGCGGCAGCTGGGGCGCCGGCGGCATCAGTGGACACGGCGTCGA
CGATGGTCATCTTGGCGCGGCGATGATGATAAGAGACCGACCAGCTGTTCCTCTTTAGCTAGTTCGTCACGAAAGCTA
GCTAGCTCGCGGTGTGTCTCGTAGTA**GTAGTAGTGCCGAAGCAGAAAG**GCAGAGGAAGTTGAAGAGAGTTAGTTTT
TAGGTTAATTTCCCCTTTGTGTGTGCGGTCGAGTGATGA**GATGACCGAGTTGGTAGCTAGG**TTTGGTAGGATTCTTTC
TTGCGGTCGATAATCCTCCCCATGTACCTTCATGCGTTGATACCACTTCACGCCGCTATATCAGAACTGAGAGAGTGAG
AGAGTAGAATCAGAGTCATATCACACAGGACGCAGATGTAGAGGAGAGTGTGTGTACGTCACCACCAGTATGCTATA
GACAGGTATTACCGACAGATCAAACATTCGTCGAGCGAGGCTACCAATAGCTGATCGATAGAGGTAGATACACATCTC
GTGCCTCACAAGAACGCGCCACGAGAGTCCCACAGGAGAATAGGCTTCTGTGAGTATGAGGACGCATAAAACGGATA
TCACGATGTCACACCCGCGTCCGCCCGGAGTAACGAGAAGCACTAGTCGATTGAGACTATTGAGGACGAACGAGACA
CACCGCGAGCTAGCTAGCTTTCGTGACGAACTAGCTAAAGAGGAACAGCTGGTCGGTCTCTTATCATCATCGCCGCGC
CAAGATGACCATCGTCGACGCCGTGTCCACTGATGCCGCCGGCGCCCCAGCTGCCGCCGCGGCGCCGGTGCAGCCCG
CGGGGAACGGGCAGACTGTGTGTGTCACCGGCGCGGCCGGGTACATCGCCTCGTGGCTCGTCAAGCTGCTGCTCGAG
AAGGGATACACTGTCAAGGGAACCGTCAGGAACCCAGATGACCCGAAGAACGCGCACCTCAAGGCGCTGGACGGCG
CCGCCGAGCGGCTGATCCTCTGCAAGGCCGACCTCCTGGACTACGACGCCATCTGCCGCGCCGTGCAGGGCTGCCAT
GGCGTCTTCCACACCGCCTCCCCGGTCACCGACGACCCGGAGCAAATGGTGGAGCCGGCGGTGCGCGGCACGGAGT
ACGTGATCAACGCGGCGGCGGAGGCCGGCACGGTGCGGCGGGTGGTGTTCACGTCCTCCATCGGCGCGGTGACCAT
GGACCCCAGCCGCGGGCCCGACGTCGTGGTCGACGAGTCGTGCTGGAGCGACCTCGAGTTCTGCAAGAAAACCAGG
AACTGGTACTGCTACGGCAAGGCGGTGGCGGAGCAGGCGGCGTGGGACGCGGCCCGGCAGCGCGGCGTGGACCTG
GTGGTGGTGAACCCGGTGCTGGTGGTGGGCCCGCTGCTGCAGCCGACGGTGAACGCCAGCATCGCGCACGTGGCCA
AGTACCTGGACGGCTCCGCGCGCACCTTCGCCAACGCCGTGCAGGCGTACGTGGACGTCCGCGACGTCGCCGACGCG
CACCTCCGCGTCTTCGAGAGCCCGCGCGCGTCCGGCCGATACCTCTGCGCCGAGCGCGTCCTCCACCGCGAGGACGTC
GTCCGCATCCTCGCCAAGCTCTTCCCCGAGTACCCCGTCCCCACCAGGTGCTCCGACGAGGTGAATCCGCGGAAGCAG
CCGTACAAGTTCTCGAACCAGAAGCTCCGGGACCTGGGGCTGGAGTTCCGGCCGGTGAGCCAGTCGCTGTACGACAC
GGTGAAGAACCTTCAGGAGAAGGGCCACCTGCCGGTGCTCGGAGAGCAGACGACGGAGGCCGACGACAAGGAGGC
GGCCCCCGCCGCCGCCGAGCTGCAGCAGGGAGGAATCGCCATCCGTGCGTGAGAGAGAGATGCCACGTACACATGA
ACAGAAAAGCAATGTTCATACAGCCCTGCCCCAGCTGTGTAAACAAACTAGTGTTTGTTCGGTGGCTGATACCCCTACC
CTAAGCTTGTTGTAACGTCATGTTCGTTCTTGTGGACTATAGTGATGAGTGAATAAAAATGGCTAATGTTCATTCATTTT
C

**Amplicon = 111 bp**

**3. SoGI - TC134289 homologue to UniRef100_Q9LLI3 Cluster: Cellulose synthase-7; n=1; Zea mays**

N8_CesA_Forward: CACCATCGGTAAGCTGGTG
N8_CesA_Reverse: AAGCCCAAATGCGAGACAG

CCGCTGACACCCTAATTCATATGGCCACAATGCTTTTGCCGTTCTTTCTTTCAGCTGTCCAACGCTGCAACGATATGGTT
TCTTGGCCCTCTTCATGTCCATCATCTTGACGAGCGTGTTGGAGCTGCGGTGGAGTGGCATCGGGATTGAGGACTGGT
GGCGCAACGAGCAGTTCTGGGTCATTGGAGGTGTGTCCGCGCACCTGTTCGCCGTGTTCCAGGGTATTCTCAAGATGA
TTGCCGGGCTGGACACCAACTTCACGGTCACGGCGAAGGCCACGGACGACACCGAGTTCGGGGAGCTGTACGTGTTC
AAGTGGACAACGGTGCTGATCCCGCCCACCAGCATCCTGGTGCTCAACCTGGTGGGCGTGGTGGCTGGCTTCTCGGC
CGCGCTCAACAGCGGCTACGAGTCCTGGGGCCCGCTCTTTGGCAAGGTGTTCTTCGCAATGTGGGTGATCATGCACCT
GTACCCGTTCCTCAAGGGTCTCATGGGCCGCCAGAACCGCACGCCCACCATCGTGGTGCTCTGGTCCGTCCTCCTCGCC

TCCGTCTTCTCCCTCCTCTGGGTCAAGATCGACCCATTCGTCGGAGGAACCGAGACCGTCAACACCAACAACTGCAACA
CAGTCATCTGCTGATT**CACCATCGGTAAGCTGGTG**TCGGGCC

**SUGIT Cellulose synthase**

CGAGAGAAAAGACCAGGCTACCAACACCACAAGAAGGCAGGGGCTATGAATGCTCTGGTCCGAGTGTCCGCTGTTCT
GACAAATGCACCTTACATTCTTAATCTTGACTGTGATCACTATGTTAACAACAGCAAAGCCGTTCGTGAAGCAATGTGC
TTCATGATGGACCCTACTGTTGGCAGAGATGTTTGCTATGTGCAATTCCCCCAGAGGTTCGATGGCATTGATCGCAGT
GATCGATATGCCAATAGGAACGTTGTGTTCTTTGATGTTAATATGAAAGGACTTGATGGCATCCAAGGCCCAGTTTAT
GTGGGAACTGGTTGTTGTTTCTATAGGCAGGCACTTTATGGTTATGGACCTCCATCTCTGCCTGCACTTCCAAAGTCTT
CAATTTGTTCGTGGTGTTGCTGCTGCTGTCCCAAGAAAAAGGTTGAAAGAAGTGAGAGGGAAATCAACAGAGACTCC
CGGCGAGAAGACCTTGAGTCTGCCATTTTTAATCTTCGTGAAATTGACAACTACGATGAGTATGAGAGGTCCATGCTG
ATCTCTCAGATGAGCTTCGAGAAATCTTTTGGGCTGTCCTCAGTCTTTATTGAATCAACTCTTATGGAGAATGGGGGCG
TCCCTGAATCTGCAAACCCATCTACCCTAATTAAAGAAGCCATTCATGTCATTAGCTGCGGATATGAAGAGAAAACTGA
ATGGGGAAAAGAGATTGGCTGGATCTATGGTTCAGTTACAGAGGATATTCTGACTGGGTTTAAGATGCAGTGCCGTG
GCTGGAGATCCATCTACTGCATGCCGGTGAGACCTGCATTCAAGGGATCAGCCCCTATCAATCTTTCCGATCGTCTTCA
CCAGGTTCTCCGGTGGGCTCTTGGTTCTGTCGAGATCTTCCTCAGTCGGCACTGCCCGCTGTGGTACGGTTACGGTGG
CGGCCGTCTGAAATGGCTCCAGAGGCTGTCCTACATCAACACCATCGTGTACCCGTTCACTTCTCTTCCTCTCGTTGCCT
ACTGTTGCCTGCCTGCCATTTGCCTGCTCACAGGAAAGTTCATTATTCCTACGCTGTCCAACGCTGCAACGATATGGTTT
CTTGGCCTCTTCATGTCCATCATCTTGACGAGCGTGTTGGAGCTGCGGTGGAGTGGCATCGGGATTGAGGACTGGTG
GCGCAACGAGCAGTTCTGGGTCATTGGAGGTGTGTCCGCGCACCTGTTCGCCGTGTTCCAGGGTATTCTCAAGATGAT
TGCCGGGCTGGACACCAACTTCACGGTCACGGCGAAGGCCACGGACGACACCGATTTCGGGGAGCTGTACGTGTTCA
AGTGGACGACGGTGCTGATCCCGCCCACCAGCATCCTGGTGCTCAACCTGGTGGGCGTGGTGGCTGGCTTCTCGGCC
GCGCTCAACAGCGGCTACGAGTCCTGGGGCCCGCTCTTTGGCAAGGTGTTCTTCGCAATGTGGGTGATCATGCACCTG
TACCCGTTCCTCAAGGGTCTCATGGGCCGCCAGAACCGCACGCCCACCATCGTGGTGCTCTGGTCCGTCCTCCTCGCCT
CCGTCTTCTCCCTCCTCTGGGTCAAGATCGACCCATTCGTCGGAGGAACCGAGACCGTCAACACCAACAACTGCAACA
CAGTCATCTGCTGATT**CACCATCGGTAAGCTGGTG**TCGGGCCGCGGCCGCCGTGTTTCTGGTGTTGCAGCCTGATGCA
GGCGGGTTACCTCTGT**CTGTCTCGCATTTGGGCTT**TTGCCCCAGGATCTGGGAGAGGTGGTATAGGTTAGCTCTGTTT
TACATCCAAGTGTTGATTGCTGTCGTCTGTATTATGAAAAGTTTTGGTGCTAAAGTGTCAAACTCGCCTCAATTGTGAG
AATGCACTGTTTAAGTGTGTGTAAATCATAGGAACGTGGGAAAAAGGAACTGAGATACACTTGCCTACCAAAAGGCA
AGATCATTTACAT

**4. SoGI -TC130010 homologue to UniRef100_A0EJ90 Cluster: Brittle stalk-2; n=1; Zea mays**

| | |
|---|---|
| N3_ BC1l5_Forward | CCTTCCACCTCTCATCTGTCG |
| N3_ BC1l5_Reverse | TATGCCCTGCCTACTCTGC |

CCCACGCGTCCGCAAGGACGCCAGGACCTTCACCTTCAGCCAGGGCTGGGCCTTCCCGCGCAAGATCTACTTCAACGG
CGACGAGTGCAAGATGCCACCGCCGGACTCCTACCCCTACCTGCCCAACGCCGCGCCCGCCGTCGCCTCGCAGCTGAT
CGTGTCCGCCGCCGCCTCAGCGTTCCTACTGGCGCTGCTCCTGGTAGCATGACCATGACCGACCAGGGCCTCCATTTCT
TCCTTTGTTTC**CCTTCCACCTCTCATCTGTCG**CCGTCTTCAATCCCATTCCCATGATCCCATCCCGTGGGCAGGGGGCA**G**
**CAGAGTAGGCAGGGCATA**TTGGTTGCCAAGACTTCAACACTTGGGTTTTGCTTCGCTTAATTGCTTCGTCAGAGGGAA
ACTGTCATGCACTGTATGTAATGTAACTTCCCAGTTGCAGGTCGTCAGATCCCAAGACAATCAGGCTGGCAGACTTTTT
GGGTCTGCAACTACTATTAGAGAGCCTGCCTGAGCGAGAGAGAGGGGGAAAAAAAGCAGACATATAGAGCATACAG
GTTTTCTGTTATTTCGTTCTGTAAACATAATGCAGATACTCTGTTCAAATGATATACGAATCAACTTACTGGACATTAGC
ATGCT

**Amplicon = 87 bp**

**SUGIT BC1l5**

GGGTCGGAGCTTGTGCTACCAGCTAGCAGCAGCCGTGGTGGTGTTACTTACGTTCTTGCAGAGGCCGCCGGCCGGCT
CGCTCATCGATCGATGGGGCTCCGCGACTCCTCCGCGCTGCTGGCTCTGGCCCTCGCCCTCGCCTACTGCTCCGTCGCA
GGTTCAATTCGTCGTCACGCCATTCCATCATCTGAAAACCAACCTTGTTTCGTTCTATCTAATGTCGATCAACGGACGAT
GCTCACCTCTCTTGTCGATCGTCGTCTTCCTCCCTGGCTGGCTGACGGCTTGCTCTCCTTGCAGTGGTGGCCTACGATCC
CCTGGACCCGAGGGGCAACATCACCATCAAGTGGGACGTGATCTCGTGGACGCCCGACGGGTACGTGGCGATGGTG
ACGATGAGCAACTACCAGATGTACCGGCACATCATGGCGCCCGGGTGGACGGTGGGGTGGTCGTGGGCCAAGAAGG
AGGTGATCTGGTCCATCGTGGGCGCGCAGGCCACGGAGCAGGGCGACTGCTCCAAGTTCAAGGCCGGCATCCCGCA
CTGCTGCAAGCGGACGCCCGCCGTGGTGGACCTGCTCCCGGGCGTGCCCTACAACCAGCAGATCGCCAACTGCTGCA
AGGCCGGCGTGGTGTCGGCGTACGGGCAGGACCAGGCGGGGTCCGTCTCCGCGTTCCAGGTCTCCGTCGGCCTCGCC
GGCACCACCAACAAGACGGTGAAGCTGCCCAAGAACTTCACCATCATGGGGCCCGGGCCAGGGTACACCTGCGGGCC
GGCGACGATCGTGCCGTCCACCGTGTACTGGACGCCTGACCACCGGCGGCGGACGCAGGCGCTCATGACGTGGACC
GTCACCTGCACCTATTCGCAGCAGCTGGCGTCCAAGTACCCGTCCTGCTGCGTCTCCTTCTCCTCCTTCTACAACGACAC
CATCGTGCCGTGCGCACGGTGCGCGTGCGGCTGCGGCCACGGCGGCCACGCCGGGCCGGGAGGGTGCATCGAGGG
GGACTCCAAGCGCGCGCTGTCGCCCGGGGTGAACACGCCGCGCAAGGACGGGCAGGCGCTGCTGCAGTGCACGCCG
CACATGTGCCCCATCCGGGTGCACTGGCACGTCAAGCTCAACTACAAGGACTACTGGCGCGCCAAGATCGCCATCACC
AACTTCAACTACCGGATGAACTACACGCAGTGGACGCTCGTGGCGCAGCACCCCAACCTGGACAACGTCACCGAGGT
CTTCAGCTTCCAGTACAAGCCGCTGCTACCATACGGCAGCATCAATGACACTGGCATGTTCTACGGGCTCAAGTTCTAC
AACGACCTGCTCATGGAGGCCGGCCCGTTCGGCAACGTGCAGTCGGAGGTGCTCATGCGCAAGGACGCCAGGACCTT
CACCTTCAGCCAGGGCTGGGCCTTCCCGCGCAAGATCTACTTCAACGGCGACGAGTGCAAGATGCCACCGCCGGACTC
CTACCCCTACCTGCCCAACGCCGCGCCCGCCGTCGCCTCGCAGCTGATCGTGTCCGCCGCCGCGTTCCTACTGGCGCTG
CTCCTGGTAGCATGACCATGACCGACCAGGGCCTCCATTTCTTCCTTTGTTTC**CCTTCCACCTCTCATCTGTCG**CCGTCTT
CAATCCCATTCCCATGATCCCATCCCGTGGGCAGGGGGCA**GCAGAGTAGGCAGGGCATA**TTGGTTGCCAAGACTTCA
ACACTTGGGTTTTGCTTCGCTTAATTGCTTCGTCAGAGGGAAACTGTCATGCACTGTATGTAATGTAACTTCCCAGTTG
CAGGTCGTCAGATCCCAAGACAATCAGGCTGGCAGACTTTTTGGGTCTGCAACTACTATTAGAGAGCCTGCCTGAGCG
AGAGAGAGGGGGAAAAAAAAGCAGACATATAGAGCATACAGGTTTTCTGTTATTTCGTTCTGTAAACATAATGCAGA
TACTCTGTTCAAATGATATACGAATC

**Amplicon = 87 bp**

## References


1 Hoang, N. V. *Analysis of genes controlling biomass traits in the genome of sugarcane (Saccharum spp. hybrids)* PhD Degree thesis, The University of Queensland, (2017).
